# A microfluidic chip for screening individual cancer cells via eavesdropping on autophagy-inducing crosstalk in the stroma niche

**DOI:** 10.1038/s41598-017-02172-7

**Published:** 2017-05-17

**Authors:** Hacer Ezgi Karakas, Junyoung Kim, Juhee Park, Jung Min Oh, Yongjun Choi, Devrim Gozuacik, Yoon-Kyoung Cho

**Affiliations:** 1 Molecular Biology, Genetics and Bioegineering Program, Sabanci University, Istanbul, 34956 Turkey; 2 Department of Biomedical Engineering, School of Life Sciences, Ulsan National Institute of Science and Technology (UNIST), Ulsan, 44919 Republic of Korea; 3 Center for Soft and Living Matter, Institute for Basic Science (IBS), Ulsan, 44919 Republic of Korea; 4 Center of Excellence for Functional Surfaces and Interfaces for Nano Diagnostics (EFSUN), Sabanci University, Istanbul, 34956 Turkey

## Abstract

Autophagy is a cellular homeostatic mechanism where proteins and organelles are digested and recycled to provide an alternative source of building blocks and energy to cells. The role of autophagy in cancer microenvironment is still poorly understood. Here, we present a microfluidic system allowing monitoring of the crosstalk between single cells. We used this system to study how tumor cells induced autophagy in the stromal niche. Firstly, we could confirm that transforming growth factor β1 (TGFβ1) secreted from breast tumor cells is a paracrine mediator of tumor-stroma interaction leading to the activation of autophagy in the stroma component fibroblasts. Through proof of concept experiments using TGFβ1 as a model factor, we could demonstrate real time monitoring of autophagy induction in fibroblasts by single tumor cells. Retrieval of individual tumor cells from the microfluidic system and their subsequent genomic analysis was possible, allowing us to determine the nature of the factor mediating tumor-stroma interactions. Therefore, our microfluidic platform might be used as a promising tool for quantitative investigation of tumor–stroma interactions, especially for and high-throughput screening of paracrine factors that are secreted from heterogeneous tumor cell populations.

## Introduction

Interactions between cancer cells and the neighboring stroma play a critical role in tumorigenesis, and an in-depth understanding of intercellular communication is of great significance for the development of novel therapeutic strategies^1–3^. Heterogeneity of tumor cells is evident, and its profound impact in clinical applications is highly recognized^4^. However, conventional tools used to study cell-to-cell interactions only deliver averaged information from a population of cells and fail to provide information on the distribution of responses reflecting the heterogeneity of individual cells.

Microfluidic devices have emerged as useful tools for single-cell analysis^5–7^. Phenotype heterogeneity^8^, paracrine secretion^9^, and DNA repair capacities with different genetic backgrounds^10^ are among the cellular properties that have been analyzed using single-cell based systems. Cell-to-cell interactions may also be studied at a single-cell level. For example, using single-cell pairing techniques, effects of cell-to-cell interaction on migration and proliferation patterns^11^ and contact-dependent organoid formation^12^ have been analyzed. In addition, the heterogeneous dynamics of CD8 T-cells during their interaction with lymphocytes have been investigated^13^. However, to the best of our knowledge, single-cell-based techniques have been rarely used for studying the interactions of tumor cells with tissues surrounding them, i.e., the stroma. Furthermore, the retrieval of individual cells for downstream molecular analyses is not straightforward but requires special tools such as photodegradable hydrogel^14^, enzymatic release of microplates^15^, microraft array^12^, or dielectrophoresis^16^.

Tumor–stroma interactions are crucial for survival, growth, and infiltration of cancer cells, as well as for metastasis and chemotherapy resistance^2^. In this study, we designed a biochip system that allows the time-course measurement of cancer cell–stroma interactions at a single-cell level. This was followed by molecular profiling of the retrieved individual cells, allowing the assessment of the correlation between phenotype distribution of intercellular interactions and their genetic bases.

In this study, MDA-MB-231 (MDA) triple-negative breast carcinoma cells were used as a tumor cell model and mouse fibroblasts expressing an autophagy marker protein called GFP-LC3, were used as a stroma model. Autophagy is an evolutionary conserved cellular stress response and recycling mechanism^17^. Recent studies indicate that autophagy in the stroma might play a key role in cancer–stroma interactions, helping to sustain tumor growth and metastasis^18–20^. In this context, it was proposed that non-protein mediators such as reactive oxygen species (ROS) and glutamine were responsible for the communication between tumor cells and stroma. However so far, the contribution of proteins and/or peptides during tumor-stroma interaction-mediated autophagy has not been studied in detail.

Here, we present a novel single-cell based screening chip system that enables quantitative analysis of tumor cell-induced autophagy in fibroblasts. The microfabricated chip consists of a custom-designed and functionalized PDMS membrane where fibroblasts cover the bottom surface only, and holes on the membrane contain entrapped individual MDA breast cancer cells. Cell-to-cell communication in the vicinity of individual holes and effects of secreted-paracrine factors was studied using this set-up.

Through proof of concept tests, we could demonstrate that TGFβ1, a cytokine that is important for tumor–stroma interactions and transdifferentiation of fibroblasts to carcinoma-associated fibroblasts (CAFs), induced autophagy in fibroblasts. Moreover, we proved that the biochip system permitted easy recovery of selected single cells, and their consequent genetic analysis was possible. Therefore, the proposed platform offers a new tool for the study of paracrine factors that mediate communication between individual tumor cells and the stromal niche and permits quantitative understanding of their genetic and phenotypic properties. Discoveries in this field might lead to the development of new diagnostic and therapeutic strategies.

## Results

### Single-cell based microfluidic chip design for monitoring autophagy in tumor-stroma crosstalk

**Figure 1:**
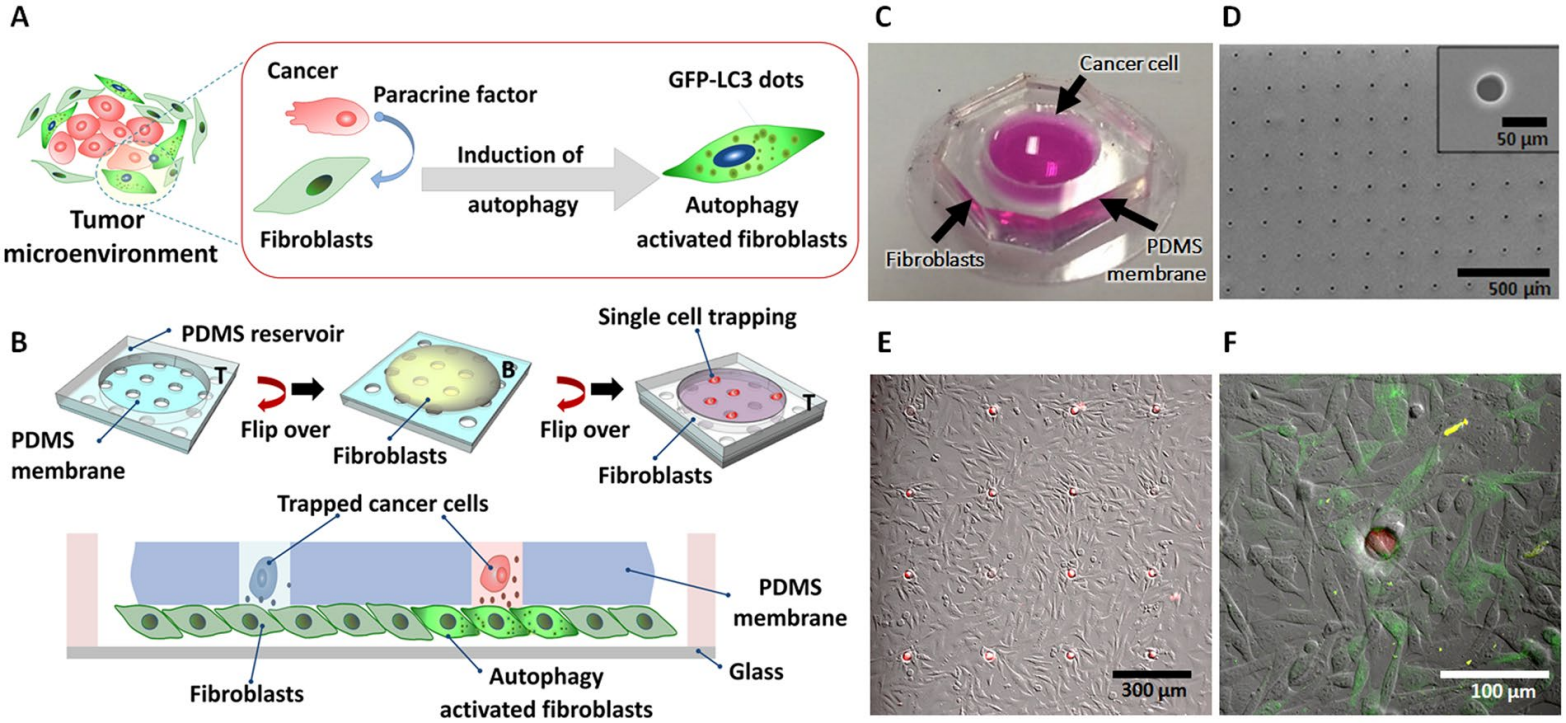
Experimental schemes and microfluidic devices for monitoring multicellular interaction between a single cancer cell and many fibroblasts in the neighbor. (**A**) In a tumor microenvironment, cancer cells communicate with neighboring fibroblasts. Autophagy induction by paracrine factors can be detected using GFP-LC3 dots quantification. (**B**) The experimental set-up that allowed the study of the interaction between the single tumor cell and fibroblasts. Fibroblasts were first cultured on the bottom side (B) of the PDMS membrane for 2 h. The PDMS membrane was turned over and attached to a PDMS coated cover-glass. Then, a single tumor cell was trapped into the holes on top side (T) via gravitational forces and agitation. (**C**) A photograph of the microfabricated biochip system. (**D**) SEM image of the membrane. The pore diameter is 30 μm (Inlet. Scale bar, 50 μm), and the center-to-center distance between the pores is 310 μm (Scale bar: 500 μm). (**E**) A 10x microscopic image of fibroblasts neighboring individual MDA cells that were trapped in the hole array (Scale bar: 300 μm). (**F**) Visualization of autophagy activation in fibroblasts during communication with trapped single MDA cells (Scale bar: 100 μm).

In a tumor microenvironment, cancer cells are involved in dynamic interactions with resident stroma cells (Fig. 1A). Recent evidence suggests that cancer cells induce autophagy in the surrounding stroma fibroblasts, and use digested materials and metabolites produced by them as a nutrient source. However, detailed mechanisms of the crosstalk have not been fully elucidated^18, 19^. Therefore, in order to study the communication between a single tumor cell and the stroma niche, we developed a porous membrane-based single-cell trapping device (Fig. 1B). In this device, GFP-LC3 (Green fluorescent protein GFP-fused to MAP1LC3 protein) transgenic immortalized MEF (mouse embryonic fibroblasts) cells modeled cancer stroma fibroblasts. In fact, GFP-LC3 serves as a commonly used tool for autophagy detection. Recruitment of GFP-LC3 to autophagic vesicles resulted in the transformation of the diffuse GFP signal into dot-like puncta, allowing detection of autophagy activation in live cells under a fluorescent microscope (Fig. 1A and B). MDA-MB-231 triple-negative breast carcinoma cells, which stably express the red fluorescent protein (RFP) (in short, MDA cells) were used as cancer cells (Fig. 1B).

The device platform was composed of a PDMS reservoir, a PDMS membrane, and a PDMS-coated thin glass (Fig. 1B and C). The fabrication process of the microfluidic chip was explained in detail in Supplementary Fig. S1 and SI Methods. As confirmed by scanning electron microscope (SEM) analyses, the membrane has approximately 800 holes with a diameter of 30 μm, which was adjusted to ideally fit a single cancer cell having a size of ranging between 10–20 μm, and the distance between adjacent holes was 280 μm (Fig. 1D). After bonding the PDMS membrane with the PDMS reservoir, the top side of the membrane was covered with a cell repellent material, i.e., 0.4% (w/v) polyethylene oxide and polypropylene oxide trioblock co-polymer (PEO-PPO-PEO), while the bottom side of the membrane was coated with 10 μg/ml of fibronectin (Supplementary Fig. S2A). Therefore, fibroblasts were entirely cultured on the bottom part of the membrane, while tumor cells settled in the membrane holes and not on the surface of the top side of the membrane (Fig. 1B). In addition, we confirmed in independent control experiments that the fibronectin coating did not leak to the top side of the membrane through the holes (Supplementary Fig. S2B and C). Furthermore, we checked that coating with PEO-PPO-PEO copolymer prevented non-specific binding of cancer cells on the surface of the PDMS membrane (Supplementary Fig. S2 D–F).

Fibroblasts were cultured on the fibronectin-coated bottom side of the PDMS membrane. Then the membrane was flipped and the device was assembled. MDA cells were introduced to the top reservoir for single cell trapping in membrane holes. A different number of MDA cells and orbital agitation velocities were tested in order to obtain optimized single cell trapping ratio and exclusion of non-trapped cells from the top side of membranes (Supplementary Fig. S3). The percentage of holes containing one and only one cell was determined in order to measure the performance of the single cell trapping ratio. Among the many conditions used, optimal single cell trapping ratio was obtained when 10^4^ MDA cells were seeded, and under 100 rpm agitation for 5 min followed by 5 times washes (Supplementary Fig. S3B and C). We also confirmed that the viability of fibroblasts was not affected (Supplementary Fig. S4A) and autophagy was not induced in GFP-LC3 fibroblasts by mere culturing on the platform (Supplementary Fig. S4B and C). As shown in Fig. 1E, under optimized conditions, individual cancer cells trapped in multiple arrays of holes in the membrane were in contact with the layer of fibroblasts cultured on the bottom side of the membrane. Upon interaction of single cancer cells trapped in pores, autophagy was induced in the neighboring fibroblasts (Fig. 1F).

### Autophagy activation by secreted paracrine factors in the tumor microenvironment

**Figure 2:**
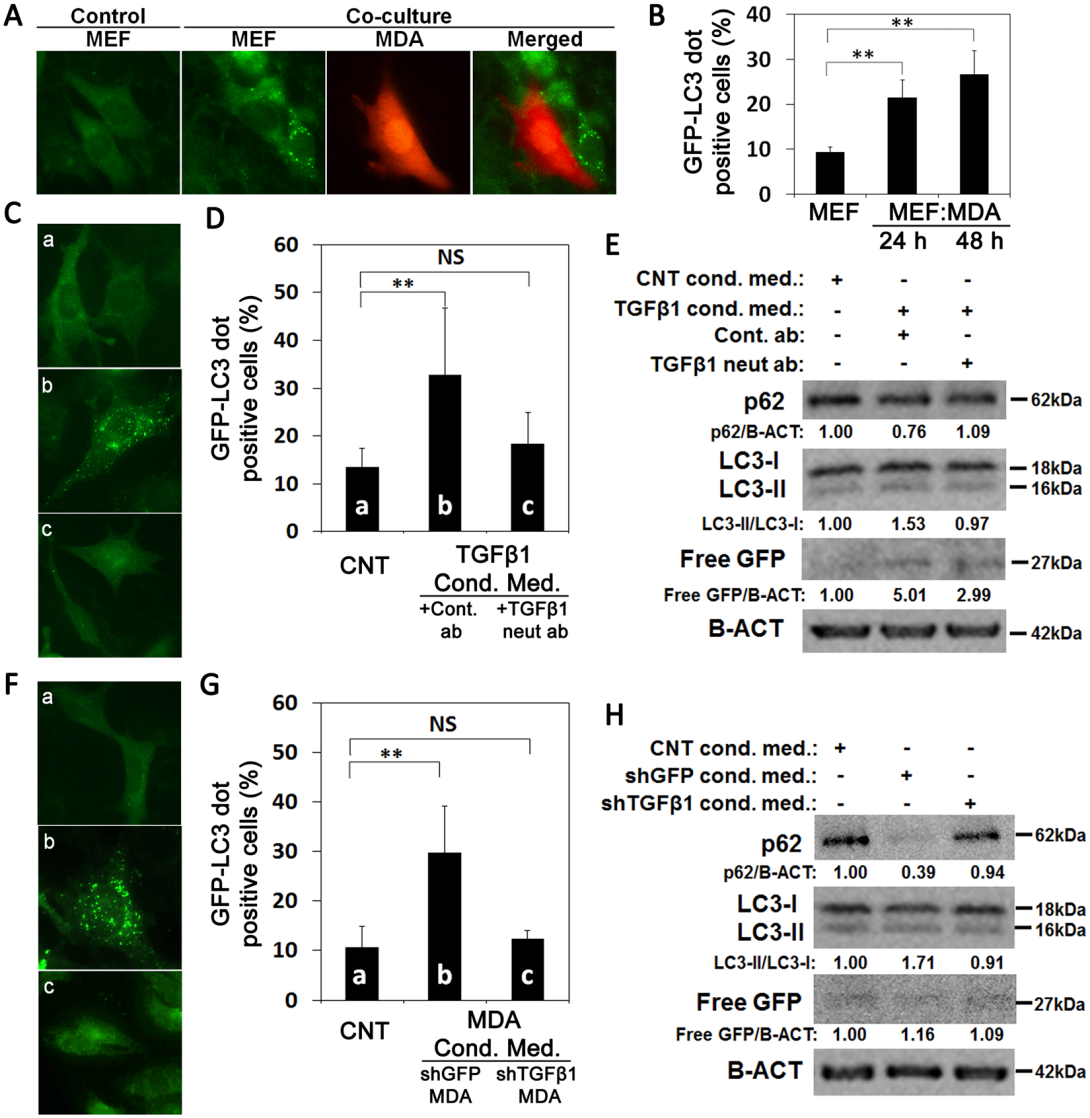
Effect of cancer cell co-culture with fibroblasts and conditioned media on fibroblast autophagy. (**A**) Co-culture of MDA-MB 231 cells (MDA) with GFP-LC3 MEFs (MEF) induced autophagy in MEFs. (**B**) MEFs that were co-cultured with MDA cells for 24 h or 48 h showed significantly higher autophagy levels compared to MEFs alone (mean ± SD of independent experiments, n = 3, **p < 0.01). (**C** and **D**) Effect of TGFβ1 neutralizing antibody (c, neut ab) or control antibody (b, cont. ab) on fibroblasts autophagy during incubation with TGFβ1 overexpressed HEK 293T cells conditioned media (cond. med.). pcDNA3 overexpressed HEK 293T cells conditioned medium was used as control (CNT) (a). The graph represents quantification of autophagy (GFP-LC3 dots) (mean ± SD of independent experiments, n = 4, NS: Non-significant, **p < 0.01). (**E**) Immunoblots of the autophagy analysis in MEFs. p62, SQSTM protein. LC3 protein, MAP1LC3. Free GFP, GFP generation from the cleavage of GFP-LC3 protein in the autolysosomes. B-ACT was used as loading control. p62/B-ACT, endogenous LC3-II/LC3-I and free GFP/B-ACT protein band densitometric analyses were performed using Image J. (**F** and **G**) Effect of conditioned media (cond. med.) from control shGFP (b) or shTGFβ1 (c) infected MDA cells on fibroblast autophagy. Conditioned medium from MEF was used as control (a). The graph represents quantification of autophagy (GFP-LC3 dots) (mean ± SD of independent experiments, n = 4, NS: Non-significant, **p < 0.01). (**H**) Immunoblot analysis of autophagy of cell extracts from MEFs that were grown in conditioned media from control shGFP or shTGFβ1 infected MDA cells. CNT, MEFs cultured in MEF conditioned medium.

When MDA cells were co-cultured with MEFs on cover slides in a 1:10 ratio and GFP-LC3 dot formation ratios were quantified, autophagy activation was clearly observed in the MEFs that were located near the MDA cells (Fig. 2A). Furthermore, the fraction of autophagy positive fibroblasts, defined as cells with more than 20 GFP-LC3 dots, increased with time (Fig. 2B). Detailed criteria for imaging and autophagic cell counting are provided in Methods.

Among the many paracrine factors, TGFβ1 was proposed to play an important role in the formation of CAF from naive fibroblasts^21^. Moreover, TGFβ1 signals contribute toward myofibroblastic cell properties during fibrosis^22, 23^ and CAF formation^24^. Studies that were published during the preparation of this manuscript also indicated that TGFβ1 could induce autophagy in fibroblasts^25, 26^. Therefore, in proof-of-concept experiments, we used TGFβ1 as a model cytokine that could mediate cancer-fibroblast interaction.

First, we confirmed TGFβ1 secretion in our system using the ELISA method. While these tests confirmed that fibroblasts also secreted TGFβ1, a significant contribution of cancer cell-derived TGFβ1 was observed in cancer cell-fibroblast co-cultures (Supplementary Fig. S5). We observed a 27.5% increase in TGFβ1 levels compared to MEF-alone when MDA breast cancer cells were co-cultured with MEFs.

To prove that TGFβ1 was indeed an inducer of autophagy, we incubated GFP-LC3 MEFs in recombinant TGFβ1–containing medium in the presence of a TGFβ1 neutralizing antibody or a control antibody. As shown in Fig. 2C and D, while autophagy induction occurred in the GFP-LC3 MEFs that were incubated in cultured medium containing HEK 293T-derived recombinant TGFβ1 and control antibody, blockage of TGFβ1 using a specific neutralizing antibody prevented autophagy activation. Moreover, under similar conditions, classical molecular markers of autophagy activation, namely endogenous LC3-I/LC3-II conversion, p62 protein degradation, as well as free GFP production from GFP-LC3 protein tests confirmed that autophagy activation was TGFβ1 dependent (Fig. 2E).

To further prove that endogenous TGFβ1 that was secreted from MDA cells was the rate-limiting cytokine for the observed autophagy activating effects, we collected media from MDA cell cultures that were infected either with lentiviruses containing a shRNA against GFP or TGFβ1 and added them onto the GFP-LC3 MEFs in culture. Off note, TGFβ1 expression levels were significantly reduced in MDA cells that were infected with the shTGFβ1 virus compared to controls (Supplementary Fig. S6). Conditioned media from control GFP shRNA (shGFP) infected MDA cells could significantly stimulate autophagy in GFP-LC3 MEFs compared to the control cells incubated with MEF-derived media. Strikingly, knockdown of the endogenous TGFβ1 in MDA cells using shRNA clearly prevented autophagy induction in fibroblasts (Fig. 2F and G). These results were also confirmed using endogenous LC3-I/LC3-II conversion, p62 protein degradation, as well as free GFP production tests (Fig. 2H). These experiments proved that TGFβ1 which was produced and secreted by the MDA cells was one of the key rate-limiting and important factors for the activation of autophagy in fibroblasts.

**Figure 3:**
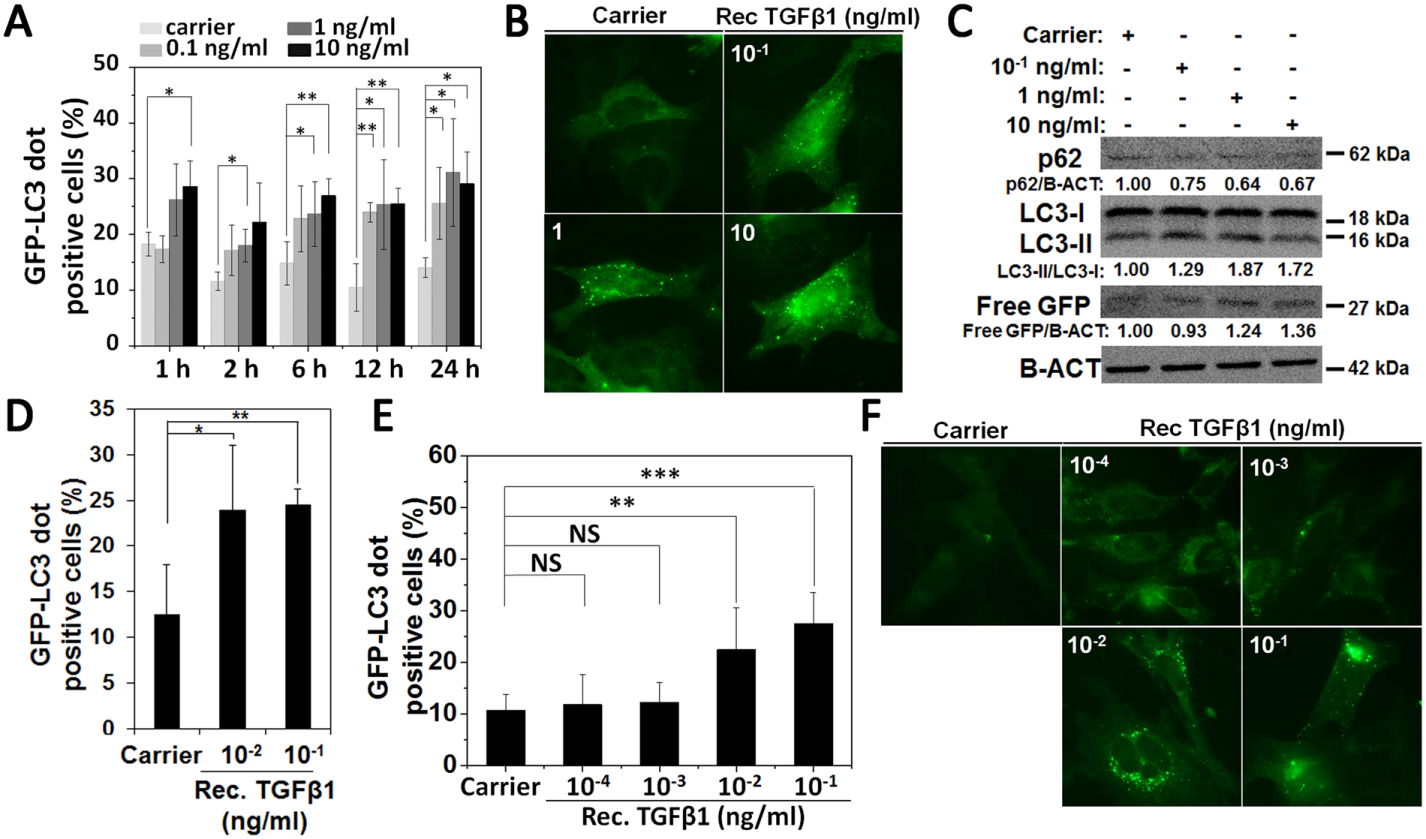
The effect of recombinant TGFβ1 on autophagy of fibroblast. (**A** and **B**) Effect of recombinant TGFβ1 (Rec. TGFβ1) on autophagy of MEFs in a dose-dependent manner (10^−1^, 1, 10 ng/ml Rec. TGFβ1). The graph represents quantification of autophagy (GFP-LC3 dots) after 1 h, 2 h, 6 h, 12 h and 24 h of incubation (mean ± SD of independent experiments, n = 4, *p < 0.05, **p < 0.01). (**C**) Immunoblot analysis for autophagy markers in MEF extracts following 24 h treatment with Rec. TGFβ1 (10^−1^, 1 and 10 ng/ml) (**D** and **E**) Effect of decreasing doses of Rec. TGFβ1 on MEF autophagy. The graph represents quantification of autophagy (GFP-LC3 dots) after 12 h of incubation (mean ± SD of independent experiments, n = 3, *p < 0.05, **p < 0.01) (**D**) and 24 h (mean ± SD of independent experiments, n = 3; NS: Non-significant, **p < 0.01; ***p < 0.001) of incubation (**E** and **F**).

In order to analyze dose and time kinetics of the cytokine, we performed GFP-LC3 dot formation assays in the presence of purified recombinant TGFβ1 protein (Rec. TGFβ1). 1 ng/ml was determined as the minimum TGFβ1 concentration that was required to induce autophagy in GFP-LC3 MEFs at 2 h and 6 h incubation times (Fig. 3A and B). Autophagy induction at concentrations under these conditions was confirmed using endogenous LC3-I/LC3-II conversion, p62 protein degradation, as well as free GFP production tests (Fig. 3C). To determine the minimum concentration of TGFβ1 that was capable of inducing autophagy, GFP-LC3 MEF cells were incubated for 12 h or 24 h with decreasing concentrations of TGFβ1. The lowest autophagy-inducing dose of TGFβ1 was determined as 10^−2^ ng/ml at 12 h and 24 h incubation time points (Fig. 3D–F).

### Numerical analysis of the diffusion of TGFβ1 secreted from a single tumor cell

**Figure 4:**
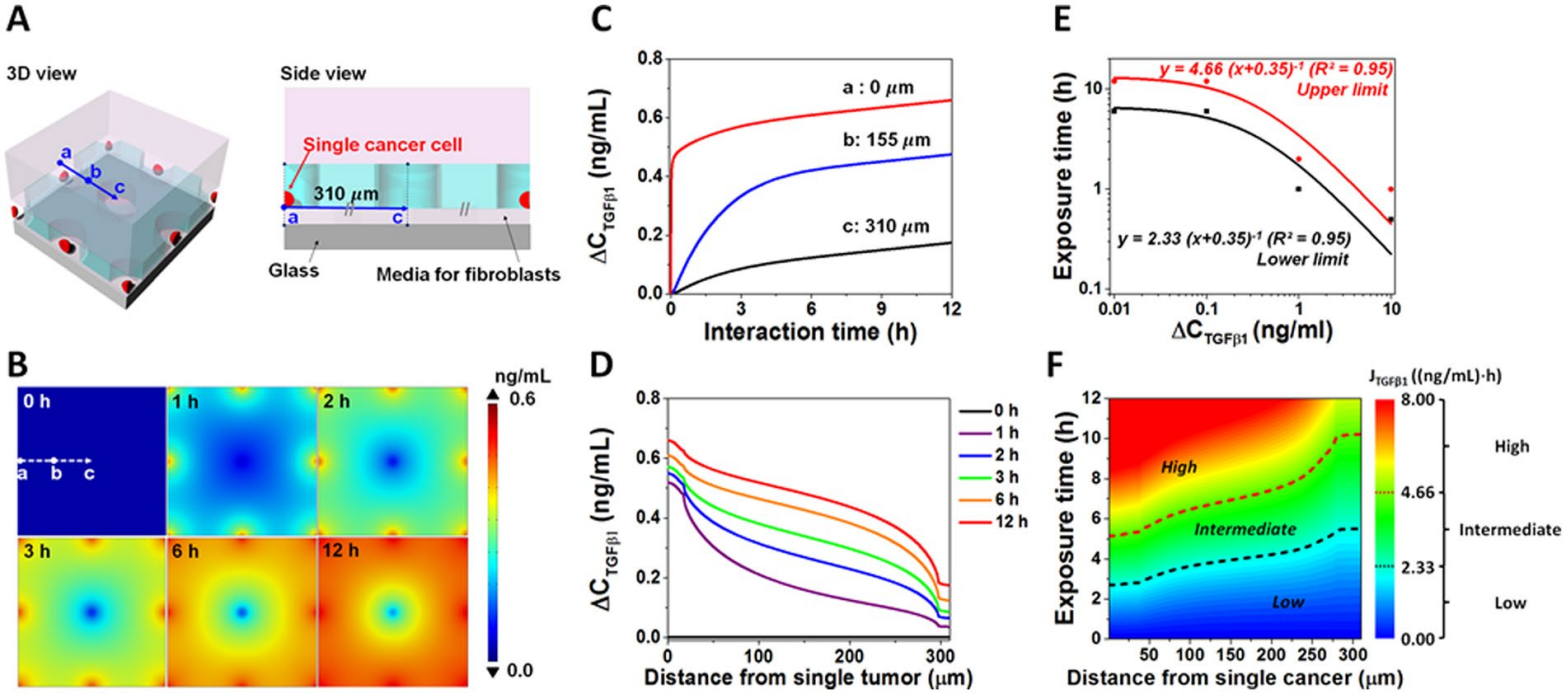
Numerical simulation of secreted TGFβ1 diffusion in the chip system. (**A**) Schematic diagram showing 3-dimensional and side views of DOI for a simulation scenario where an empty hole is situated near eight neighboring holes occupied by cancer cells. (**B**) Distributions of the concentration difference of TGFβ1 (${{\rm{\Delta }}C}_{\text{TGF}\beta 1})$ in the fibroblast region with respect to time. (**C**) Time evolution of ${{\rm{\Delta }}C}_{\text{TGF}\beta 1}$ at selected positions: (a) the center of the hole containing a single cancer cell, (b) the middle position between an empty hole and a tumor-occupied hole, and (c) the center of the empty hole. (**D**) Distribution of ${{\rm{\Delta }}C}_{\text{TGF}\beta 1}$ along the path (a-c) from a cancer cell-occupied hole to an empty hole for various time. (**E**) Hyperbolic curve fitting (solid lines) to determine exposure condition. The minimum amount of TGFβ1 exposure for highly probable autophagy activation was determined as the upper bound (red symbols) and the maximum amount of TGFβ1 exposure that is unlikely to induce autophagy was determined as the lower bound (black symbols). (**F**) Contour plot of the TGFβ1 exposure (${{\rm{J}}}_{\text{TGF}{\rm{\beta }}1}$) in (distance, exposure time)-space, where the numerical simulation was conducted. Based on ${{\rm{J}}}_{\text{TGF}\beta 1}={\int }_{0}^{{\rm{T}}}({{\rm{\Delta }}C}_{\text{TGF}\beta 1}+{\rm{b}}){\rm{d}}{\rm{t}}$, the exposure condition space can be divided into high, intermediate, and low probable regimes for autophagy activation. The optimized exposure time, which is sufficiently short so as not to affect autophagy induction via TGFβ1 diffusion from neighbor tumors and sufficiently long to induce autophagy activation at their own position, can be decided by the contour plot.

We conducted numerical simulations in order to optimize the exposure time for autophagy in experiments. The exposure time should be chosen to prevent cross-talk between holes of the membrane. Schematic descriptions of the simulation are shown in Fig. 4A. We solved a time-dependent diffusion equation for TGFβ1 using COMSOL Multiphysics®. We assumed that an empty hole is surrounded by the neighbor cancer cells. As the array of holes in the system was periodic, a single periodic unit (represented by the continuous line a–c in Fig. 4A) was taken as the domain of interest (DOI) for simulation. And, the symmetric condition was applied to the boundaries.

The geometrical dimensions, including hole diameter, distance between neighboring holes, and space between the biochip membrane and glass slide, were identical to the real experimental platform. Concentration of TGFβ1 that was secreted from MDA cancer cells was around 250 pg/ml following 24 h of incubation (^27^ and our observations). Using this value and time point, the secretion rate was calculated as 4.88 × 10^−14^ mol/m^2^∙s when the cancer cell size was assumed as 11 µm (measured by average diameters of trypsinized 100 MDA cells). The diffusion coefficient (D) of TGFβ1 in the reservoir was calculated as 6.4 × 10^−11^ m^2^/s, by using the equation of D = 1.72 × 10^−8^ (M.W.)^−0.552^ (^28^) and the molecular weight (M.W.) of TGFβ1 as 25 kDa. Furthermore, the media between the membrane and glass was assumed to be porous (${{\rm{N}}}_{i}=-\,{D}_{i,eff}\nabla C=-\,\frac{\varepsilon }{\tau }D\nabla C,\varepsilon :{\rm{porosity}}\,{\rm{and}}\,\tau :{\rm{tortuosity}})\,$since it was packed with fibroblasts. We determined basal concentration (*C*_0_) of TGFβ1 in the DMEM medium supplemented with various FBS concentrations (1~10% (v/v)) using an ELISA assay (Supplementary Fig. S5A). When ELISA results were corrected according to cell numbers on chips (66,000 cells in 300 µl medium), TGFβ1 amount secreted from MEFs after 6 h of culture was calculated as 105.93 pg/ml. Basal concentration of TGFβ1 (*C*_0_) was considered as the sum of TGFβ1 that comes from 3% FBS containing media (266.72 pg/ml) and MEFs in culture (105.93 pg/ml) (Supplementary Fig. S5). The corresponding molar density is 1.50 × 10^−8^ mol/m^3^.

TGFβ1 concentration difference profile (${\rm{\Delta }}{C}_{TGF\beta 1}=C-{C}_{0}$) around an empty hole (center) that is surrounded by eight cancer cell-containing hole is shown in Fig. 4B. Figure 4C represents the time evolution of ${\rm{\Delta }}{C}_{TGF\beta 1}$ at specified positions (a, b and c points) (Fig. 4A). ${\rm{\Delta }}{C}_{TGF\beta 1}$ near holes, that are occupied with a tumor cell (a), rises steeply at the beginning and then increases gradually to reach to approximately 0.66 ng/ml at 12 h, meanwhile the concentration at the empty hole (c) increases slowly and does not reach 0.18 ng/ml even after 12 h. Figure 4D shows the distribution of ${\rm{\Delta }}{C}_{TGF\beta 1}$ along the path (a-c distance in µm) in different time points.

According to our experimental results, a net 10^−2^ ng/ml TGFβ1 concentration was required to induce significant levels of MEF autophagy in our system at 12 h (Fig. 3D and E); and the same concentration did not induce autophagy after 6 h incubation under similar conditions (Supplementary Fig. S7). Taking these 6 h and 12 h data into consideration, concentration of TGFβ1 that was required for autophagy induction in MEFs was modeled as a function of time (Fig. 4E and F). As shown in Fig. 4E, the minimum amount of TGFβ1 exposure for highly probable autophagy activation was fitted as the upper bound (red symbols) and the maximum amount of TGFβ1 exposure that is unlikely to induce autophagy was fitted as the lower bound with a hyperbolic function ($T=A\cdot {({\rm{\Delta }}{C}_{TGF\beta 1}+b)}^{-1}$). The parameter of *A* corresponds to the accumulated amount of TGFβ1 in constant secretion condition and *b* is the fitting parameter. The contour plot of exposure amount for TGFβ1 (${J}_{\text{TGF}{\rm{\beta }}1}$), which is divided into high, intermediate, and low probable regimes for autophagy activation, was drawn by integrating $\,{\rm{\Delta }}{C}_{TGF\beta 1}$ with respect to exposure time (${J}_{\text{TGF}{\rm{\beta }}1}={\int }_{0}^{T}({\rm{\Delta }}{C}_{\text{TGF}{\rm{\beta }}1}+b)dt$) and the distance from single cancer cell (Fig. 4F). Optimized exposure time was decided in the light of these results. 6 h exposure time was long enough to induce high to intermediate levels of autophagy in MEFs around a defined tumor cell (a to b: 0 to 155 µm). Yet, at this time point, autophagy in MEFs around neighboring holes was still at low levels (b to c: 155 to 310 µm). Therefore, 6 h time point was chosen and used in the biochip experiments.

### Single cell-level monitoring of autophagy in fibroblasts interacting with a cancer cell

In the light of tumor–stroma autophagy model, we wanted to confirm that autophagy activation in response to tumor-secreted TGFβ1 in MEFs followed a gradient. In order to reduce heterogeneity in the system, we first tried to improve control vs TGFβ1 signal using chloroquine (CQ, lysosomal pH modifier) or rapamycin (Rapa, an mTOR inhibitor and autophagy inducer). But, there was no improvement (Supplementary Fig. S8). We then created monoclones of both GFP-LC3 MEF cells and MDA cancer cells. These monoclones were similar to parental cells with respect to their TGFβ1 secretion, and autophagic response to TGFβ1 treatment and starvation (Supplementary Fig. S9). In the biochip system, MEF monoclone cells were cultured alone or with single MDA monoclone cells captured in holes. Images of autophagy activation were taken from 3 different “region of interests” depending on the distance from the center of a hole. Cells that were found at a 50 μm (S), 100 μm (M), 155 μm (L) radial distance from a hole were evaluated. Percentage of GFP-LC3 dot positive cells in these 3 regions were quantified in a cumulative manner (Supplementary Fig. S10). Here, GFP-LC3 dot positivity in MEFs followed a clear gradient on biochips. MEFs that were closer to tumor cell-occupied holes had higher autophagic activity, compared to those that were farther away. However, lowest standard deviation levels and more homogenous autophagic response were obtained when autophagy activation in cells that were within 155 μm (L) radial distance were quantified. As expected, MEFs around empty holes did not activate significant levels of autophagy. Therefore, autophagy activation in biochips followed a gradient and was not random. Activation in empty holes most probably reflected basal autophagic activity in the cells. In the light of these results, we decided to evaluate autophagy activation in MEFs at a 155 μm radial distance from holes.

**Figure 5:**
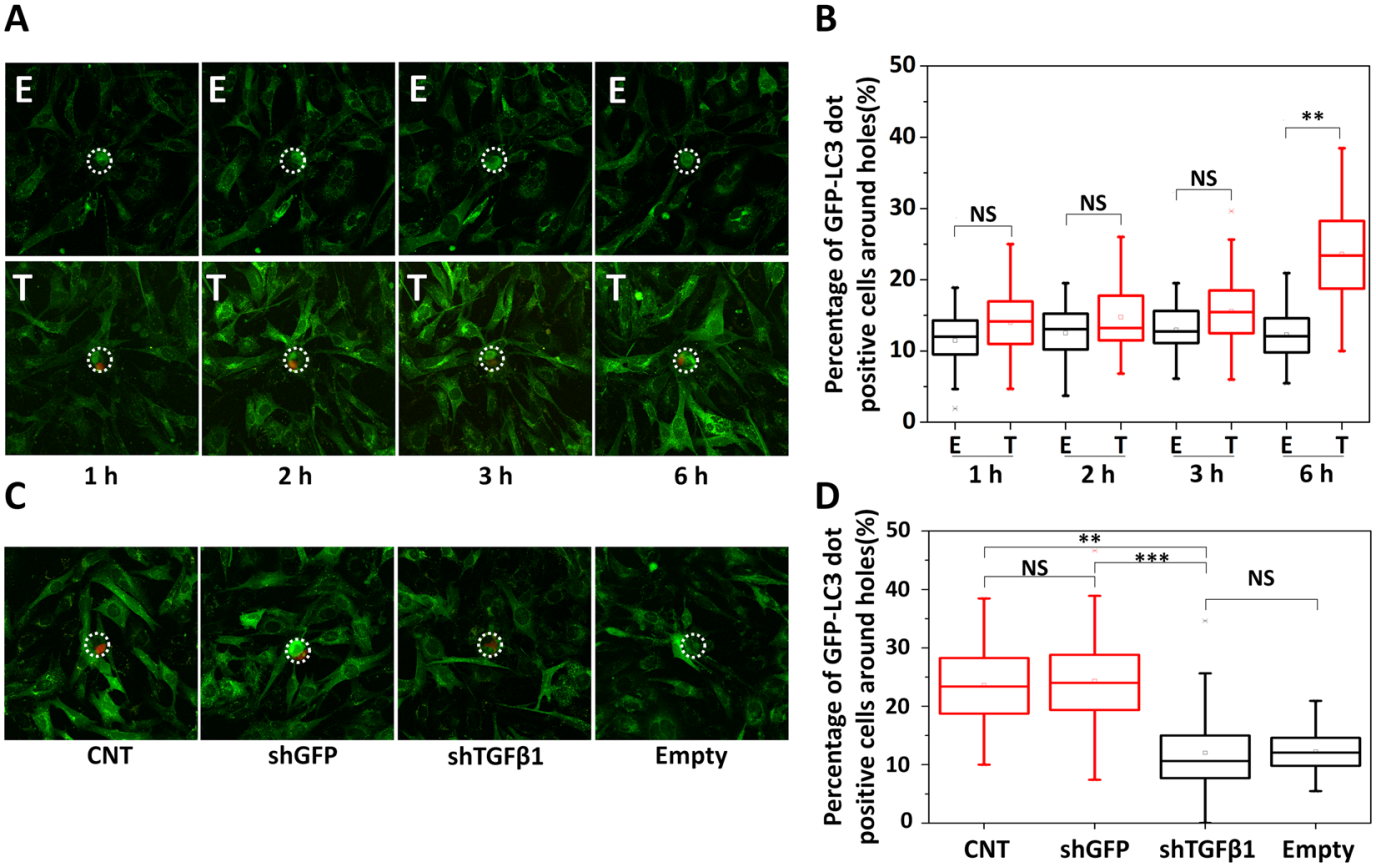
Single cell-level monitoring of autophagy in fibroblasts interacting with individual cancer cells. (**A**) Live cell images of dynamics of autophagy activation in fibroblasts that are near an empty hole (E) or a hole with a trapped single tumor cell (T). (**B**) Box plot representation of GFP-LC3 dot quantification of fibroblasts near an empty hole (E) or a hole with a tumor cell (T) (1 h, 2 h, 3 h and 6 h co-culture). After 6 h of intercellular interactions, autophagy near holes with trapped tumor cells (Number of holes = 68) could well be differentiated from those near empty holes (Number of holes = 65). (**C**) Images of autophagy activation in MEFs near a hole with a wild-type MDA cell (CNT) (Number of holes = 68), shGFP infected MDA cell (shGFP) (Number of holes = 89), shTGFβ1 infected MDA cell (shTGFβ1) (Number of holes = 84) and empty holes (Number of holes = 65). (**D**) Quantification of autophagy activation in MEFs shown in C. MEFs near shTGFβ1 infected MDA cells (shTGFβ1) had lower autophagy levels (comparable to empty holes (Empty)) compared to those near wild-type (CNT) or shGFP infected MDA cells (shGFP). Images were analyzed by Imaris software (3 independent experiments, p-value was calculated by results of each set, NS: Non-significant, **p < 0.01, ***p < 0.001).

Next, we performed time kinetics experiments of cells in biochips. We checked autophagy activation in MEFs on biochips at 1 h, 2 h, 3 h and 6 h. We observed that the most significant autophagy activation was obtained after 6 h of co-culture (Fig. 5A and B). A live cell imaging video of real-time dynamics of autophagy activation in fibroblasts were also recorded (Supplementary Movie S1). Following optimization of the autophagy detection system on biochips, the role of TGFβ1 in cancer cell-stroma interaction was tested. MDA cells were infected with control shRNA (shGFP) or shTGFβ1 constructs and GFP-LC3 MEF autophagy induction levels were quantified. In line with our results in *in vitro* cell culture dishes, TGFβ1 knockdown in MDA cells (Supplementary Fig. S11) did significantly attenuate autophagy activation in MEFs on biochips (Fig. 5C and D).

### Facile retrieval of single tumor cells and downstream molecular analysis

**Figure 6:**
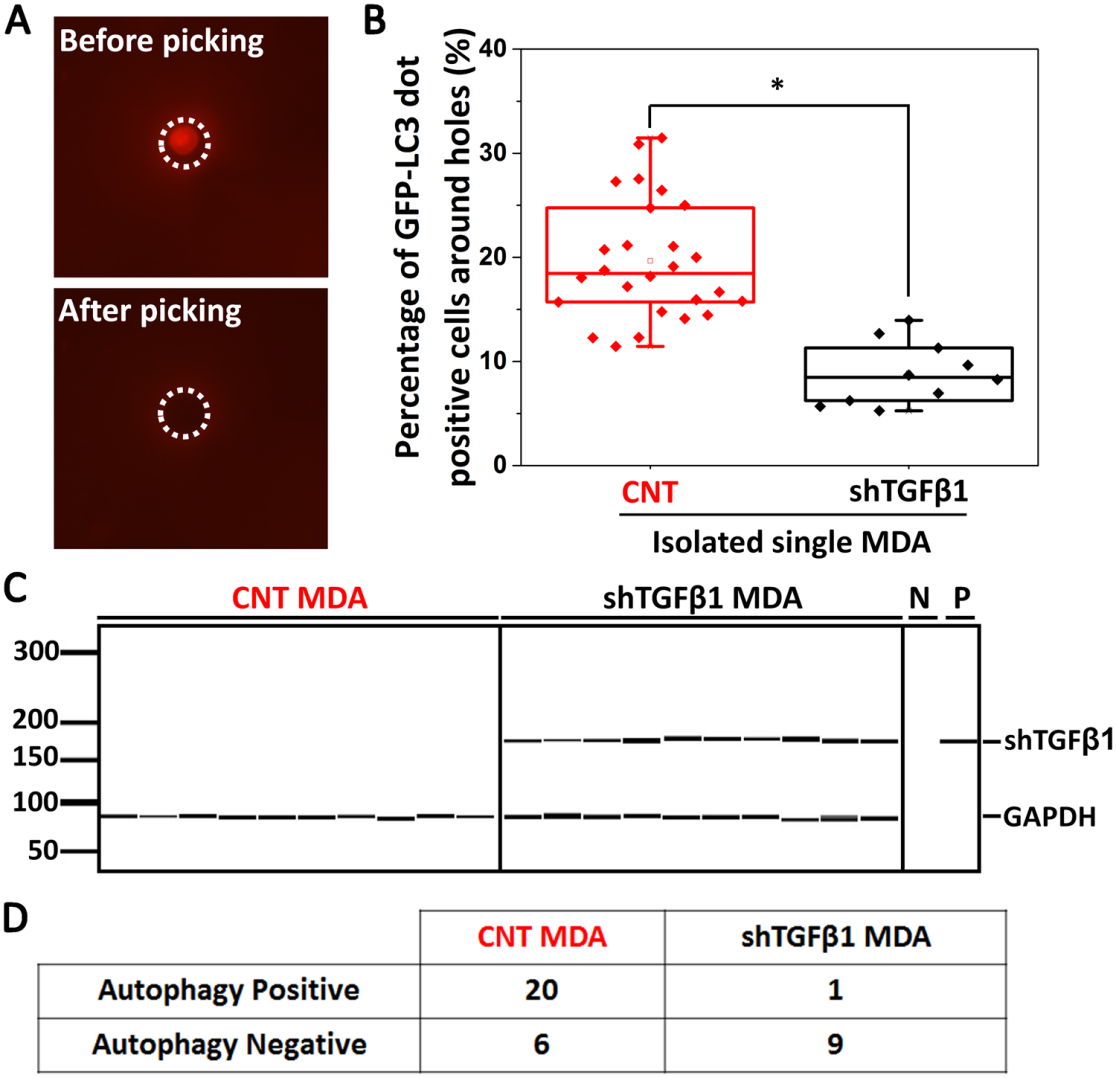
Retrieval and characterization of single tumor cells. (**A**) Using a single cell-picking device, individual cancer cells were retrieved and used for further characterization. (**B**) Box plots depicting percentage of autophagic, GFP-LC3 dot positive, MEFs (more than 15 dots/cell) around (155 µm diameter) single MDA cancer cell-containing randomly selected holes. Number of holes = 36. PCR analysis for the genomic DNA of single cancer cells was performed to confirm the presence or absence of the shTGFβ1 vector. Then, GFP-LC3 dot positivity was evaluated and results were depicted as shown in the graph. (**C**) Representative PCR results showing shTGFβ1 positivity or negativity of MDA cells that were analyzed in B. 10 representative amplification results from wild-type MDA cells (CNT MDA) and shTGFβ1 MDA cells were shown. N represents negative control without template and P represents positive control using the shTGFβ1 vector (3 independent experiments, *p < 0.05). (**D**) Summary of the results in B and C.

In order to demonstrate that the proposed platform could identify and isolate autophagy-inducing single cancer cell clones, we performed the following blind tests by using a mixture of wild-type MDA cells and shTGFβ1 infected MDA cells. We performed the single cell analysis of the MDA shTGFβ1 cells after viral infection to decide the ratio of cells in the mixture. As shown in Supplementary Fig. S12, only 8 out of 20 (40%) single MDA cells actually show the shTGFβ1 band although we could obtain 80% infection efficiency. Therefore, a mixture of wild type MDA and shTGFβ1 MDA cells with 1:4 ratio was used for blind test. After 6 h of co-culture between cancer cells and fibroblasts on biochips, GFP-LC3 dot status of MEFs were documented. Regardless of their autophagy status, single MDA tumor cells at their center were pinpointed at random, and collected from the chips using a microscope-based single-cell picking device (Fig. 6A). Genomic DNA isolation from the picked cells was performed. Using primers that are specific for the shRNA vector, we were able to PCR amplify and identify the TGFβ1 shRNA sequence in the vector. PCR results confirmed that out of 36 randomly selected single cancer cell clones, 10 cells contained the shTGFβ1 sequence in their DNA. When blindly taken cell pictures were analyzed retrospectively, most MEF cells with less or no autophagy were around holes with MDA cells containing the shTGFβ1 sequence (Fig. 6B and C).

The cut-off value was for the percentage of GFP-LC3 dot positive MEF cells near a hole was previously decided as 15% based on the capacity of the biochip system to differentiate the CNT MDA cells from shTGFβ1 containing MDA cells with a highest accuracy (82.23% in Fig. 5D). In other words according to our criteria, to label a hole as autophagy positive, the hole should have around it (155 µm radius area) more than 15% of the MEFs containing >20 GFP-LC3 dots per cell. Among 36 MDA cells which were randomly picked-up from 3 independent chips, pictures of MEF cells that were around 21 holes showed higher autophagy signals than the cut-off value, so they were considered as autophagy positive (Fig. 6B and C). Among cancer cells retrieved from these 21 positive holes, 20 cells were indeed CNT cells and only one cell was shTGFβ1 positive. Remaining 15 holes were autophagy negative. 9 of 15 cells were indeed shTGFβ1 positive MDA cells (Fig. 6B and C). whereas 6 cells were identified as CNT MDA cells. Figure 6D summarizes the results of these tests.

Therefore, the sensitivity (or, true positive rate) which is defined as the percentage of the holes, whose signal were higher than the cut-off value among total number of holes containing wild type tumor cells, was 77% (20/26). On the other hand, the specificity (or, true negative rate), which is defined as the percentage of the holes whose signal were lower than the cut-off value among total number of holes containing shTGFβ1 MDA cells, was 90% (9/10). As a result, we can differentiate CNT MDA and shTGFβ1 MDA cells based on their single-cell level capability of inducing autophagy in neighboring fibroblasts with 81% accuracy. These results suggest that our platform can be used in shRNA-based screens of cancer cells and allow a high-throughput study of the mechanisms of autophagy activation in fibroblasts in a tumor microenvironment.

## Discussion

In this work, a single cell-based screening platform was developed, in which the degree of autophagy activation induced by the intercellular communication between tumor cells and fibroblasts was used as criteria. In these chips, trapping of single tumor cells in the membrane holes of the microfluidic chip followed by co-culture with adjacent fibroblasts was possible. In proof-of-concept tests, we demonstrated in cell culture dishes and on microfluidic chips the effect of TGFβ1 on paracrine autophagy activation in fibroblasts. While this manuscript was in preparation, in line with our results, Cai *et al*. confirmed autophagy-inducing effects of TGFβ1 on fibroblasts and in the formation of CAF phenotype in a tumor microenvironment^29^.

To the best of our knowledge, this is the first study to demonstrate at a single cell level that autophagy in tumor stroma, can actually be used as a tool to screen for autophagy mediators in individual cancer cells. Therefore, the technique has the potential to reveal key mediators secreted from tumor cells by performing a genome-wide analysis of target cancer cells after autophagy analysis in fibroblasts.

Importantly, the microfluidic platform was suitable for the retrieval of single tumor cells of interest, allowing genomic, transcriptomics and even metabolomics and determination of clonal backgrounds of individual cells. As such, the platform is suitable for large-scale unbiased omics screens.

Future large-scale studies using this microfluidic chip platform might provide novel insights into tumor biology, and allow characterization of key molecular mediators of autophagy during tumor–stroma communication and other biological and pathological phenomena. Indeed, our platform has the potential to be applied to different cellular systems (e.g. tumor cell-macrophage, tumor cell-stem cell couples etc.) and reveal further information on tumor-stroma interactions at a single cell level.

## Methods

### Cell culture

Mouse embryonic fibroblasts (MEFs) from GFP-LC3 transgenic^30, 31^ was immortalized as polyclones using the SV40 whole genome. RFP-expressing MDA cells were created by infecting MDA cells with pRSI9-U6-(sh)-UbiC-TagRFP-2A-Puro empty shRNA library viruses and selecting them with 2 μg/ml puromycin containing medium for 30 days. HEK 293T cells were cultured at 37 °C with 5% CO_2_ and in DMEM (Sigma-D5671) -supplemented with 10% (v/v) fetal bovine serum (FBS; Biochrom KG, S0115), L-glutamine (Biological Industries, 03-020-1B), and 100 U/ml penicillin/streptomycin (Biological Industries, 03-031-1B). MDA-MB-231 cells and GFP-LC3 MEFs were cultured in DMEM-supplemented with 10% FBS, L-glutamine, 100 U/ml penicillin/streptomycin, and 1X MEM non-essential amino acid solution (Gibco, 11140-035).

### Lentiviral transduction

Lentiviruses were produced by the co-transfection of pRSI9-U6-(sh)-UbiC-TagRFP-2A-Puro plasmid with helper plasmids psPAX2 and pMD2.G or by transfection of shTGFβ1/shGFP (kindly gifted by Tamer Önder) with helper plasmids pCMV-VSVG and pCMV-dR8.2 into HEK 293T cells. Culture media were harvested at 48 h and 72 h after transfection, and used to infect cells or stored at −80 °C. To create stable cell lines, MDA cells were infected at 60% confluence for 24 h with lentiviral supernatants diluted 1:1 with fresh culture medium in the presence of 5 µg/ml polybrene (Sigma, H9268) or 5 ng/ml protamine sulfate (Sigma, P4505). 24 h later, fresh culture medium was added. Infected cells were selected for 4 weeks in culture media containing 2 µg/ml puromycin.

### Conditioned culture media preparation

Culture media of MDA cells and TGFβ1 or pcDNA3 transfected HEK 293T cells were changed with DMEM medium, containing L-glutamine, 100 U/ml penicillin and streptomycin and 3% FBS. After 72 h incubation, conditioned culture media were collected and concentrated using an Amicon^®^ Ultra-4 centrifugal filter device (Millipore, UFC800324). In order to avoid autophagy activation by impoverished media, conditioned media were concentrated and diluted with fresh culture media before adding onto MEFs.

### Co-culture of MDA cells and fibroblasts

GFP-LC3 MEFs (1 × 10^5^ cells/ml) were seeded on cover slides. After 16 h, MDA cancer cells were added onto MEFs in order to obtain a cancer cell: fibroblast ratio of 1:10. Cells were examined 24 h or 48 h later.

### Recombinant protein incubations and antibody neutralizations

Conditioned media from TGFβ1 overexpressing HEK 293T cells or culture media containing recombinant TGFβ1 protein (Sigma Cat #: H8541) were used right away or mixed with 30 µg/ml TGFβ1 neutralizing antibody (R&D, Catalog #: AB293) or control serum (rabbit serum: Sigma, R9133). Following rotation at 4 °C for 2 h, fresh media (DMEM; 10% FBS; L-glutamine; 100 U/ml Pen/Strep.; 1X nonessential aminoacids) was added. GFP-LC3 MEFs were cultured in this mixed media for 24 h and then analyzed.

### GFP-LC3 dot analyses

Following indicated treatments, GFP-LC3 MEFs were fixed in 4% paraformaldehyde (Sigma, 158127) for 20 min, washed with PBS, mounted and inspected under 60x magnification using a BX60 fluorescence microscope (Olympus, BX60). Dots per cell were counted (at least 100 cells) and basal autophagy threshold was determined as 20 GFP-LC3 dots per GFP-LC3 MEF under non-treated conditions. At least 150 cells per condition were counted and autophagy was expressed as a percentage of dot positive cells (>20 dots) to the total cell population.

### Analysis of basal and induced autophagy in microfluidic chip system

Fibroblasts were seeded in the microfabricated PDMS membrane platform, the bottom of which was coated with 10 µg/ml fibronectin. The medium in the reservoir was changed after 16 h of fibroblasts culture. After 6 h of interaction with cancer cells, the autophagy was measured by counting the percentage of fibroblasts with more than 20 GFP-LC3 dots under different conditions. In total, around 50 fibroblasts were analyzed under a confocal microscope for each condition by using a Plan Apo λ 40x objective lens (NA 0.95) (A1R, Nikon).

### RNA isolation and real-time PCR

Total RNA was extracted using the PureLinkTM RNA mini kit (ThermoFisher Scientific) according to the manufacturer’s instructions. cDNA was reverse transcribed from total RNA using the SuperScript® VILO™ cDNA Synthesis Kit (ThermoFisher Scientific). Real-time RT-PCR analyses were performed as previously described using SYBR Green an QuantiStudio6 thermal cycler (Life Technologies)^32^. PCR reactions were: Initial cycle of 95 °C, 10 min, then PCR reactions of 40 cycles of 95 °C for 15 s and 60 °C for 1 min. A thermal denaturation protocol was used to generate the dissociation curves for verifying the amplification specificity. Changes in mRNA levels were quantified using the 2-ΔΔCT method using GAPDH (glyceraldehyde-3-phosphate dehydrogenase) mRNA as control. The following primers were used during the study: TGFβ1 primers 5′-ACTGCAAGTGGACATCAACG-3′; 5′-TGCGGAAGTCAATGTACAGC-3′; GAPDH primers 5′-ATGGGTGTGAACCATGAGAA-3′; 5′-GTGCTAAGCAGTTGGTGGTG-3′.

### Immunoblotting analyses

Immunoblotting was performed as previously described^33^. Briefly, protein extraction was performed with a RIPA buffer (50 mM TRIS-HCl pH 7.4, 150 mM NaCl, 1% NP40, 0.25% Na-deoxycholate) supplemented with a complete protease inhibitor cocktail (Roche, 04-693-131-001) and 1 mM phenylmethylsulfonyl fluoride (PMSF; Sigma-Aldrich, P7626). Cell extracts were separated in 15% SDS-polyacrylamide gels and transferred to a nitrocellulose membrane. Following blockage in 5% nonfat milk in PBST (3.2 mM Na_2_HPO_4_, 0.5 mM KH_2_PO_4_, 1.3 mM KCl, 135 mM NaCl and 0.05% Tween 20, pH 7.4) for 1 h at RT, membranes were incubated in 3% BSA-PBST solutions containing primary antibodies (ab): anti-SQSTM1/p62 ab (BD Transduct. Lab, 610832, 1:1000), anti-LC3B ab (Sigma, L7543, dilution 1:1000), anti-GFP (Roche, 11814460001, dilution 1:1000) and anti-B-ACTIN ab (Sigma-Aldrich, A5441, dilution 1:7500). Then, secondary mouse or rabbit antibodies coupled to horseradish peroxidase (anti-mouse: Jackson ImmunoResearch Laboratories, 115035003; anti-rabbit: Jackson ImmunoResearch Laboratories, 111035144, dilutions 1:10,000) were applied in 5% milk/PBST for 1 h at RT, and protein bands were observed with chemiluminescence technique.

### Live cell analyses

To study the kinetics of autophagy activation a confocal microscope (A1R, Nikon) equipped with a motorized X-Y stage and a live cell chamber maintaining CO_2_ at 5% and the temperature at 37 °C was used. Images were taken using a Plan Apo λ 40x objective (NA 0.95). GFP-LC3 MEFs and MDA cells were captured at wavelengths 488.0 and 561.5 nm, respectively. Images (1,024 X 1,024 pixels) were automatically taken at 20 positions every 15 min during 6 h of interaction.

### Retrieval of single tumor cells and single cell genomic DNA PCRs

Individual tumor cells were retrieved using a Kuiqpick micromanipulator (NeuroInDx). To withdraw a single tumor cell, a capillary with a diameter of 20 µm was positioned over membrane holes. Using optimized vacuum duration and power, the trapped single tumor cell was removed in a small volume of medium. Isolated cells were released into individual tubes and stored in lysis buffer for single cell gene analyses. After retrieval, genomic DNA was isolated using the REPLI-g Single Cell Kit (Qiagen, Valencia, CA) according to manufacturer’s recommendations. For PCR, following primers were used: Forward (5′-GACTATCATATGCTTACCGT-3′) and reverse (5′-GTGGATGAATACTGCCATT-3′) primers for the shTGFβ1 vector, and forward (5′-ATGGGTGTGAACCATGAGAA-3′) and reverse primers (5′-GTGCTAAGCAGTTGGTGGTG-3′) for GAPDH with reaction parameters 95 °C for 10 min (1 cycle); 95 °C for 45 s, 57 °C for 30 s, 72 °C for 90 s for 35 cycles; and 72 °C for 3 min (1 cycle). PCR products were analyzed using a Labchip GX touch bioanalyzer (PerkinElmer).

### Statistical analyses

Statistical analyses were performed using student’s two-tail t-test. Data were represented by means of ±SD of ≥3 independent experiments. Values of p < 0.05 were considered as significant.

## Electronic supplementary material

Supporting Information

Supplementary video 1
