# Supplementary material for: A microfluidic chip for screening individual cancer cells via eavesdropping on autophagy-inducing crosstalk in the stroma niche
Source: Sci Rep. 2017 May 17;7:2050. doi: 10.1038/s41598-017-02172-7 (PMC5435728; doi:10.1038/s41598-017-02172-7)
Supplement: Supplementary file 1 — Supporting Information [file 41598_2017_2172_MOESM1_ESM.pdf]

Supporting Information

A microfluidic chip for screening individual cancer cells via eavesdropping on autophagy-inducing crosstalk in the stroma niche

Hacer Ezgi Karakas[a, 1], Junyoung Kim[b, 1], Juhee Park[c], Jung Min Oh[c], Yongjun Choi[b,c], Devrim Gozuacik[a, 2], Yoon-Kyoung Cho[b, c, 2]

Methods

Design and fabrication of the PDMS porous membrane

The PDMS porous membrane was fabricated by PDMS (Sylgard 184, Dow Corning, Midland, MI, USA) injection into the gap between a surface-treated PDMS replica and glass (Fig. S1). The PDMS replica was fabricated using photolithography and soft lithography. A negative photoresist (SU-8-2025, Microchem, Newton, MA) was coated on a 6” silicon wafer at 1,750 rpm for 30 s (JSPD, JD Tech) (Fig. S1A), and a photoresist layer was patterned by photolithography (6 min soft baking at 95 °C, 170 mJ/cm^2 exposure energy, 6 min post exposure baking at 95 °C, and IPA & DI water rinse, MA-6, SussMicroTech) (Fig. S1B). For facile release of the PDMS replica, the photoresist master on the silicon wafer was silanized by treatment with trichloro (1H, 1H, 2H, 2H–perfluorooctyl) silane (Sigma Aldrich) in a vacuum desiccator (Bel-Art Scienceware) for 24 h. PDMS (base to curing agent ratio of 5:1) was cast onto the photoresist master and cured for 2 h at 65 °C (Fig. S1C). The released PDMS replica (Fig. S1D) and glass slide were treated by reactive ion etching (Labstar, TTL) with 50 sccm CHF_3, 150 W for 100 s and 50 sccm CHF_3, 15 W for 10 s, respectively (Fig. S1E). The treated surfaces faced each other and were clamped

together by magnets. Then, PDMS (5:1) was filled via capillary action (Fig. S1F). After curing for 2 h at 65 °C, the PDMS membrane was released easily with 70% ethanol (Fig. S1G). The membrane was 50 μm thick and consists of 800 holes with 30 μm diameter, separated by the center to center distance of 310 μm. The dimensions of the pores were verified by SEM measurement (SNE 4500M, SEC corp).

Design and fabrication of single cell screening platform.

The membrane was first assembled with a 4 cm-thick PDMS reservoir with an opening window of 11 mm diameter, using oxygen plasma with 60 W plasma power and 50 sccm O_2 for 1.5 min (CUTE, Femto science) (Fig. S1I). PDMS spin coating was employed to prepare the bottom substrate that faces the bottom side of the membrane where the fibroblasts are attached and cultured; the PDMS spin coating was conducted on cover glass at 2500 rpm for 5 min. The thickness of the coated PDMS layer was measured using a surface profiler (P6, KLA Tencor); the thickness was controlled to 8 μm to prevent the cancer cells from escaping a hole and migrating to other holes. After polymerization at 65 °C for 2 h, the center part (11 × 11 mm) was cut and removed to make space for fibroblast culture media.

Surface treatment of PDMS membrane with fibronectin and PEO-PPO-PEO solution

The device was thoroughly sterilized with 70% ethanol and UV for 1 h. After sterilization of the device, the bottom part of the membrane was coated with 10 μg/ml fibronectin (Sigma, F1141) for 30 min at room temperature and washed with PBS. A 0.4% w/v PEO-PPO-PEO copolymer (Pluronic F-108, Sigma Aldrich) was coated on the top part of the membrane for 30 min, after

which the membrane was washed with PBS. In order to check the fibronectin coating status, 10 μg/ml HiLyte-488 tagged fibronectin solution was coated on the bottom side of the PDMS membrane for 30 min, after which the membrane was washed with PBS. A fluorescence image test was conducted using a confocal microscope (Zeiss) to observe the fibronectin coating status. To check the effect of the PEO-PPO-PEO coating, the percentage of tumor cells remaining on the PDMS membrane was measured after 5 times of washing with media. Fluorescence images of RFP tumor cells were obtained using an IX-71 inverted microscope (Fig S2) (Olympus).

Monolayer of fibroblasts on the microfluidic device

Fibroblasts were resuspended at 6.6×10^4 cells in 200 μL of the medium. Cells were seeded to the bottom side of the membrane and allowed to attach for 2 h in the incubator. Medium was removed and the surface was washed with DMEM. Then, the reservoir with fibroblasts was flipped to the original position and assembled with the substrate, which contained 200 μL of a DMEM medium. The medium filling the reservoir could perfuse cells through membrane holes. After 14 h, fibroblasts covered the bottom part of the membrane. Seeded single tumor cells did not actively migrate through the holes during the experiments.

ELISA Assay

TGFβ1 concentration was in culture media was analyzed using ELISA kit (R&D, Cat #: DB100B) according to manufacturer's recommendation (Fig. S5). TGFβ1 concentration in media containing 1-10% FBS was measured in the absence of cells. TGFβ1 concentration in the presence of 6.0×10^4 MEF or MDA cells in 200 μL of medium containing 3% FBS in 48-well plate. 6.0×10^3 MDA cancer cells were seeded in co-cultures. After 6 h, cultured media were

collected and analyzed. Average of two independent set of experiments were shown in Fig. S5A and B. In shTGFβ1 knock-down system, 1.0×10^5 were seeded in 2 ml of medium containing 3% FBS in 6-well plate. Cells were infected with shGFP/shTGFβ1 viruses together with 5μg/ml polybrene. After 16 h, cells were washed and media were changed to 2 ml medium containing 3% FBS. After 72 h, media were collected and analyzed. Result of three independent experiments were shown in Fig. S11.

Single cell trapping experiments with MDA cells

When fibroblasts reached confluence, suspended tumor cells were loaded to the PDMS reservoir, and single cells were allowed to be trapped in the holes via horizontal agitation (Neo Shaker, AS ONE). Then, the platform was kept in the incubator for 25 min. allow in the trapped cells to settle. Untrapped tumor cells were removed by washing 5 times with DMEM medium. The trapping ratio was calculated as the number of holes with tumor cells divided by the total number of holes in the membrane. To determine the optimum conditions for single cell trapping, number of tumor cells input (5×10^3-2×10^4 cells) and the agitation velocity (0–200 rpm) were tested (Fig. S3).

Video

Supplementary video 1: Live cell imaging of GFP-LC3 MEFs near an empty hole and a hole with single tumor cell (MDA cells) from 1 h to 6 h are shown. Images were taken every 30 min (Scale bar: 50 μm).

Supplementary Figure legends

Figure S1: Fabrication procedure for the PDMS porous membrane. (A) Deposit the photoresist on the silicon wafer. **(B)** Make a pattern using photolithography. **(C)** Add the pre-cured PDMS and cure. **(D)** Peel off the PDMS master. **(E)** Perform RIE treatment with CHF_3. **(F)** Inject the pre-cured PDMS into the gap between the PDMS master and glass. **(G)** Release the PDMS membrane. **(H)** Fabricate an 11 mm-diameter PDMS reservoir. **(I)** Attach the PDMS membrane with the PDMS reservoir.

Figure S2: The effect of surface coating on the PDMS membrane. (A) HiLyte488 fibronectin coating on the bottom side of the membrane. Before coating, the membrane did not show any fluorescent signal. The coated side of the membrane showed green fluorescence between and around the holes. **(B)** The fibronectin coating on the bottom side of the membrane did not affect the top surface, as is confirmed by the z-stack confocal image analysis of a hole. **(C)** The bottom side of the membrane showed the HiLyte488 fibronectin coating while the top surface did not. **(D)** Compared to the uncoated side, the coated side had a reduced number of cancer cells attached to the area between the holes. **(E)** Quantification of the experiments shown **D** (n=3).

Figure S3: Optimization of trapping efficiency for MDA cells in the microfluidic chip system with a different number of cancer cells and orbital shaking velocities. (A) Visualization of fibroblasts and trapped MDA cells in the microfluidic chip system under 20x. **(B)** Trapping efficiency of cancer cells with a different number of cancer cells and quantification of the experiment (mean±SD of independent experiments, n=3). **(C)** Trapping efficiency of cancer cells with different orbital shaking velocities in the microfluidic chip system and quantification of the experiment (mean±SD of independent experiments, $*p < 0.05$, n=3). Even though the student t-test does not show statistically significant difference among different conditions we tried, we found the shaking velocity of 100 rpm and 1×10^4 input number of cells provide relatively more reproducible data and used the condition in the follow-up experiments.

Figure S4: The effect of the microfluidic chip on biocompatibility and the basal level of autophagy in fibroblasts. (A) In order to perform a live/death assay, PI staining was performed for the cells cultured in glass and on fibronectin-coated PDMS membrane. Cell viability was not affected by the system with time (mean±SD of independent experiments, n=3, NS: Non-significant). **(B)** Compared to the control case when fibroblasts were seeded onto the glass slide, fibroblasts seeded into the microfluidic chip does not show elevated autophagy levels. **(C)** Quantification of the experiment **B** (mean±SD of independent experiments, n=3, NS: Non significant).

Figure S5: Analysis of TGFβ1 concentration. (A) TGFβ1 concentration (pg/ml) was analyzed in different percentages (1-10%) FBS containing media (average of two independent experiments). **(B)** TGFβ1 concentration (pg/ml) in the media of MDA cancer cells, MEFs or co-

cultures (Culture in 3% FBS containing medium for 6 h). (average of two independent experiments).

Figure S6: Analysis of TGFβ1 in MDA cells at mRNA level. Compared to control case; MDA cells infected with control shRNA (shGFP), TGFβ1 levels are significantly reduced in shTGFβ1-infected MDA cells (mean±SD of independent experiments, n=3, ***p<0.001).

Figure S7: The effect of recombinant TGFβ1 on autophagy of fibroblast. (A and **B)** Effect of decreasing doses of Rec. TGFβ1 on MEF autophagy (10^{-2}, 10^{-1}, 1 and 10 ng/ml Rec. TGFβ1). The graph represents quantification of autophagy (GFP-LC3 dots) after 6 h of incubation (mean±SD of independent experiments, n=4, NS: Non-significant, *p<0.05, **p<0.01)

Figure S8: Autophagy analysis of MEFs in the presence or absence of chloroquine (CQ, 0.1 µM or 1 µM) or Rapamycin (Rapa, 2 nM or 20 nM) with or without recombinant TGFβ1 protein (1 ng/ml). CNT, no addition of CQ or Rapa. Carrier, 4mM HCl, 0.1% BSA.

Figure S9: Characterization of MDA monoclone cancer cells and GFP-LC3 MEF monoclone. (A) Analysis of TGFβ1 secretion from MDA poly or monoclones (5F9 monoclone was shown) (average of two independent experiments). Cells were cultured in 3% FBS containing media for 6 h. Supernatant of the cells were analyzed using the ELISA assay. **(B)** Microscopy of GFP-LC3 dots in MEF poly or monoclones (4F3 monoclone was shown). Cells were treated with 1 ng/ml recombinant TGFβ1 for 6 h or starved for 2 h in order to induce autophagy. **(C)** Autophagy markers were analyzed in MEF poly or monoclones (4F3) using immunoblotting technique. Cells were treated with 1 ng/ml recombinant TGFβ1 for 6 h or

starved for 2 h in order to induce autophagy. B-ACT was used as loading control. Densitometric analysis of the bands was performed using Image J software.

Figure S10: Autophagy analysis in biochips. Autophagy in MEFs was analyzed in areas of 50 μm (S), 100 μm (M), 155 μm (L) diameter from the center of empty or single cancer cell-captured holes (mean±SD of independent chip experiments, $^{**}p < 0.01$, n=3).

Figure S11: Analysis of TGFβ1 mRNA and secreted protein levels in with shGFP (control) or shTGFβ1 virus-infected MDA cells after 72 h. (A) mRNA level of TGFβ1 was analyzed using qPCR (mean±SD of independent experiments, $^{***}p < 0.001$, n=3). **(B)** Secreted TGFβ1 concentration (pg/ml) in the media (mean±SD of independent experiments, $^{*}p < 0.05$, n=3).

Figure S12: Analysis of MDA cells 72 h after infection with lentiviruses containing shTGFβ1. Only 8 out of 20 randomly selected MDA cancer cells contained shTGFβ1 sequence in their genome although our viral infection efficiency was near 80%.

Figure S1

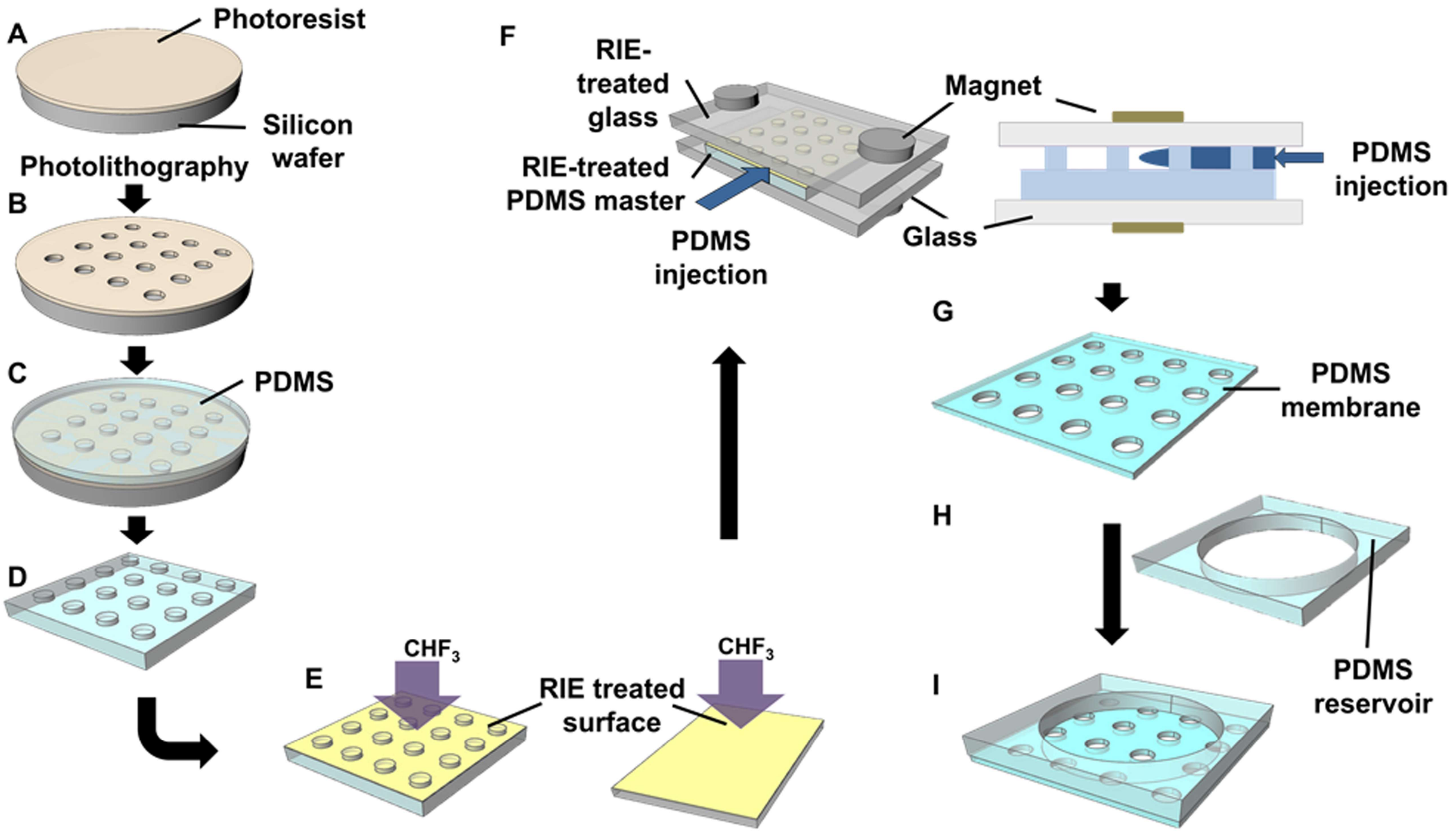
A
Photoresist
Silicon wafer
Photolithography
B
C
PDMS
D
E
CHF$_3$
RIE treated surface
CHF$_3$
F
RIE-treated glass
RIE-treated PDMS master
PDMS injection
Magnet
Glass
PDMS injection
G
PDMS membrane
H
I
PDMS reservoir

Figure S2

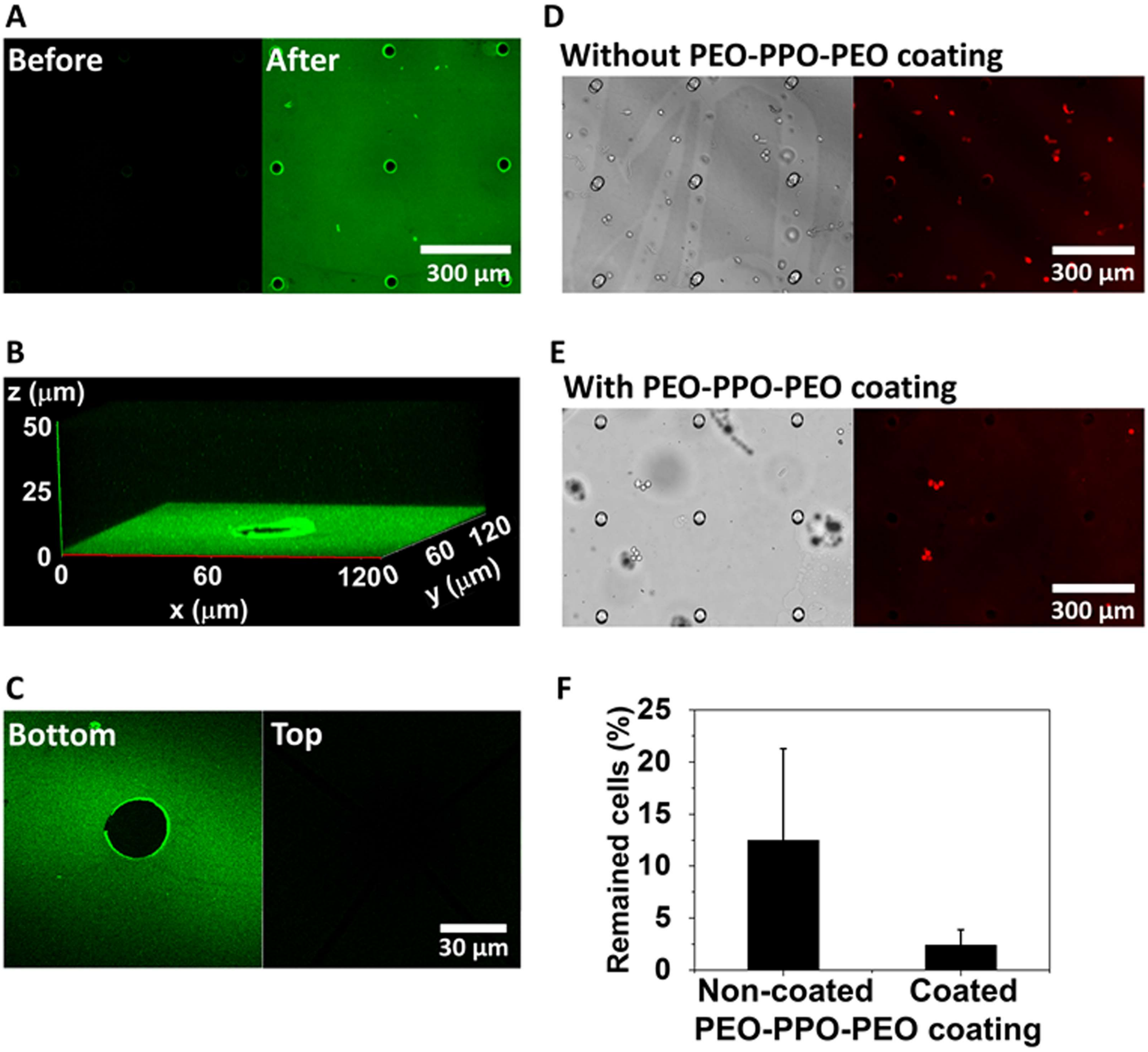
A
Before
After
300 μm
B
z (μm)
50
25
0
0
60
1200
x (μm)
60
120
y (μm)
C
Bottom
Top
30 μm
D
Without PEO-PPO-PEO coating
300 μm
E
With PEO-PPO-PEO coating
300 μm
F
Remained cells (%)
0
5
10
15
20
25
Non-coated
Coated
PEO-PPO-PEO coating

Figure S3

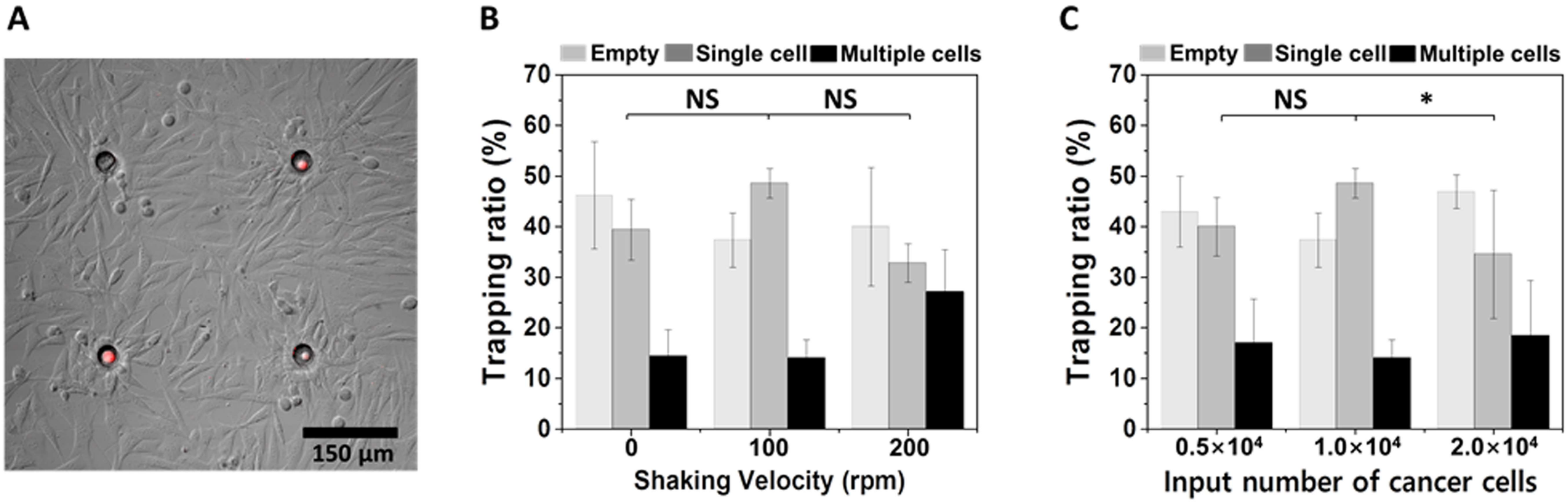
A
150 µm
B
Empty
Single cell
Multiple cells
NS
NS
Trapping ratio (%)
0
10
20
30
40
50
60
70
0
100
200
Shaking Velocity (rpm)
C
Empty
Single cell
Multiple cells
NS
*
Trapping ratio (%)
0
10
20
30
40
50
60
70
0.5×10⁴
1.0×10⁴
2.0×10⁴
Input number of cancer cells

Figure S4

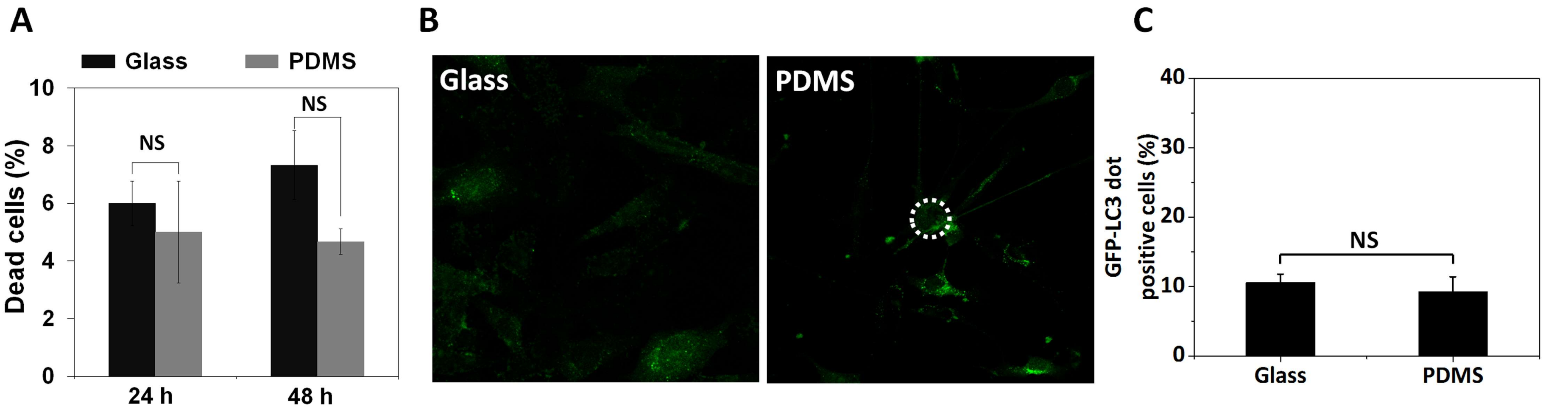
A
Glass
PDMS
10
8
6
4
2
0
Dead cells (%)
NS
NS
24 h
48 h
B
Glass
PDMS
C
40
30
20
10
0
GFP-LC3 dot positive cells (%)
NS
Glass
PDMS

Figure S5

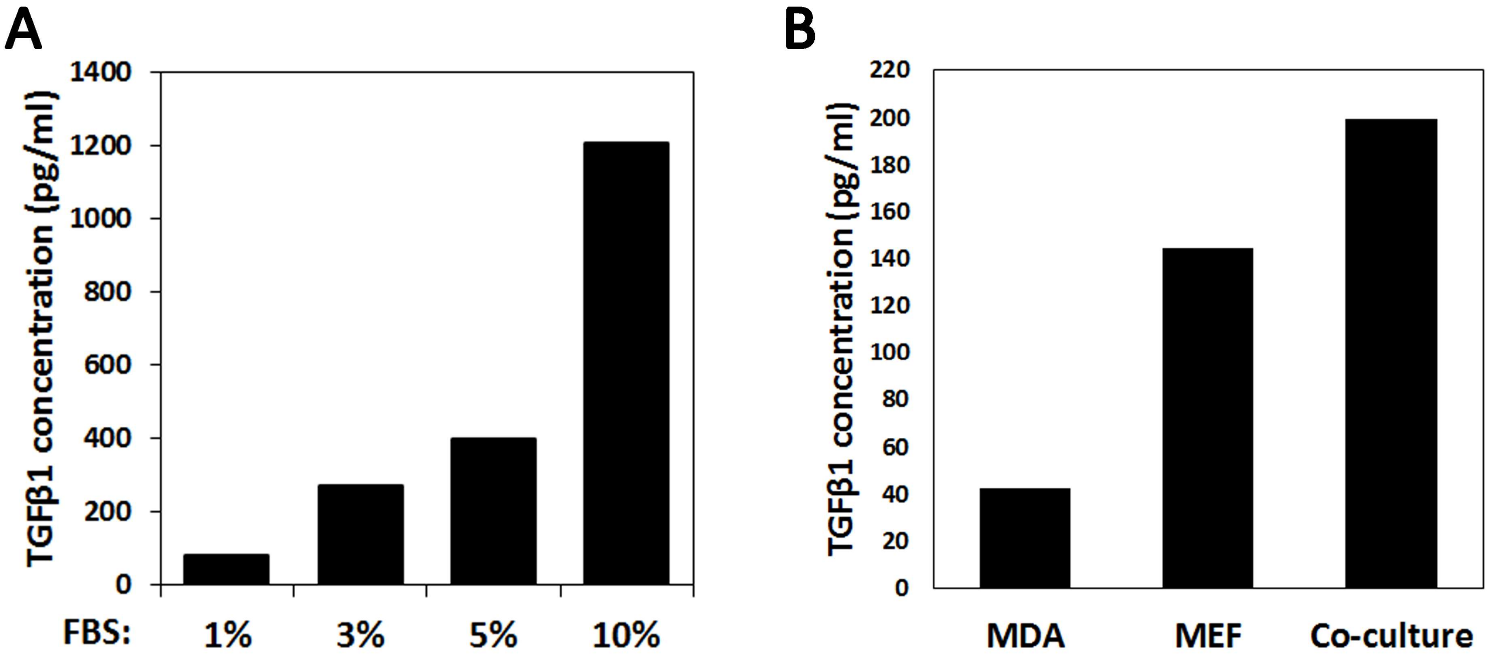
A
TGFβ1 concentration (pg/ml)
1400
1200
1000
800
600
400
200
0
FBS:
1%
3%
5%
10%
B
TGFβ1 concentration (pg/ml)
220
200
180
160
140
120
100
80
60
40
20
0
MDA
MEF
Co-culture

Figure S6

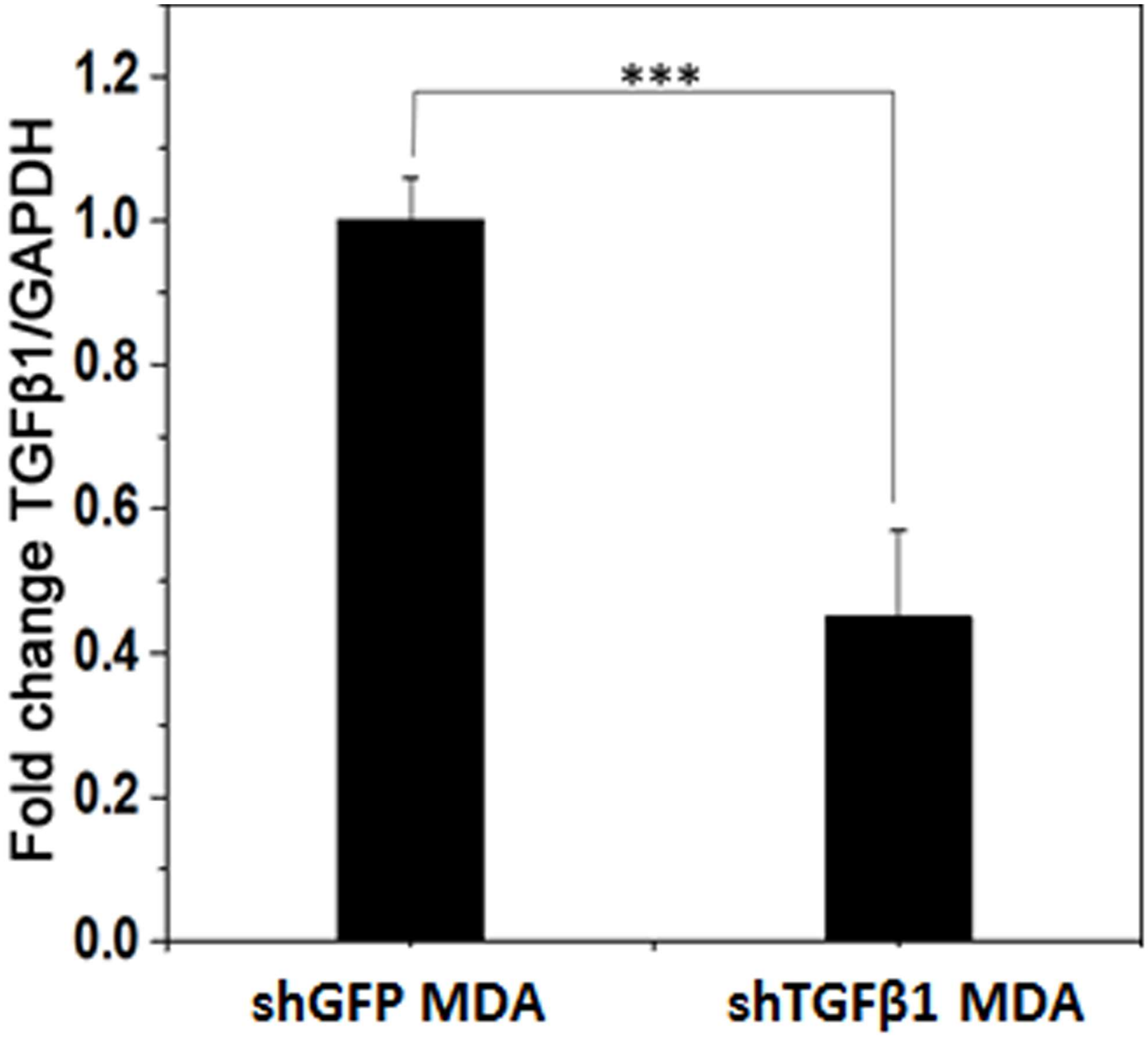
Fold change TGFβ1/GAPDH
1.2
1.0
0.8
0.6
0.4
0.2
0.0
***
shGFP MDA
shTGFβ1 MDA

Figure S7

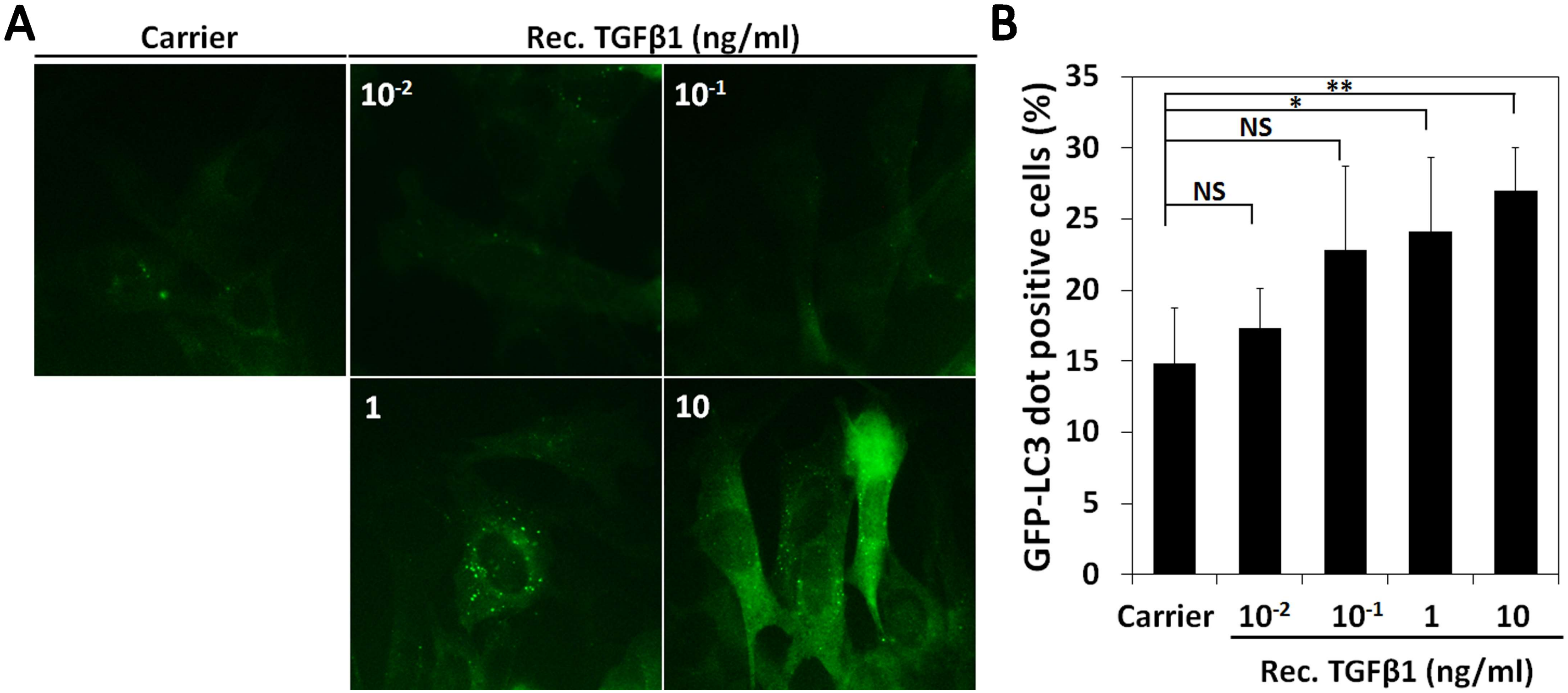
A
Carrier
Rec. TGFβ1 (ng/ml)
10-2
10-1
1
10
B
GFP-LC3 dot positive cells (%)
35
30
25
20
15
10
5
0
**
*
NS
NS
Carrier
10-2
10-1
1
10
Rec. TGFβ1 (ng/ml)

Figure S8

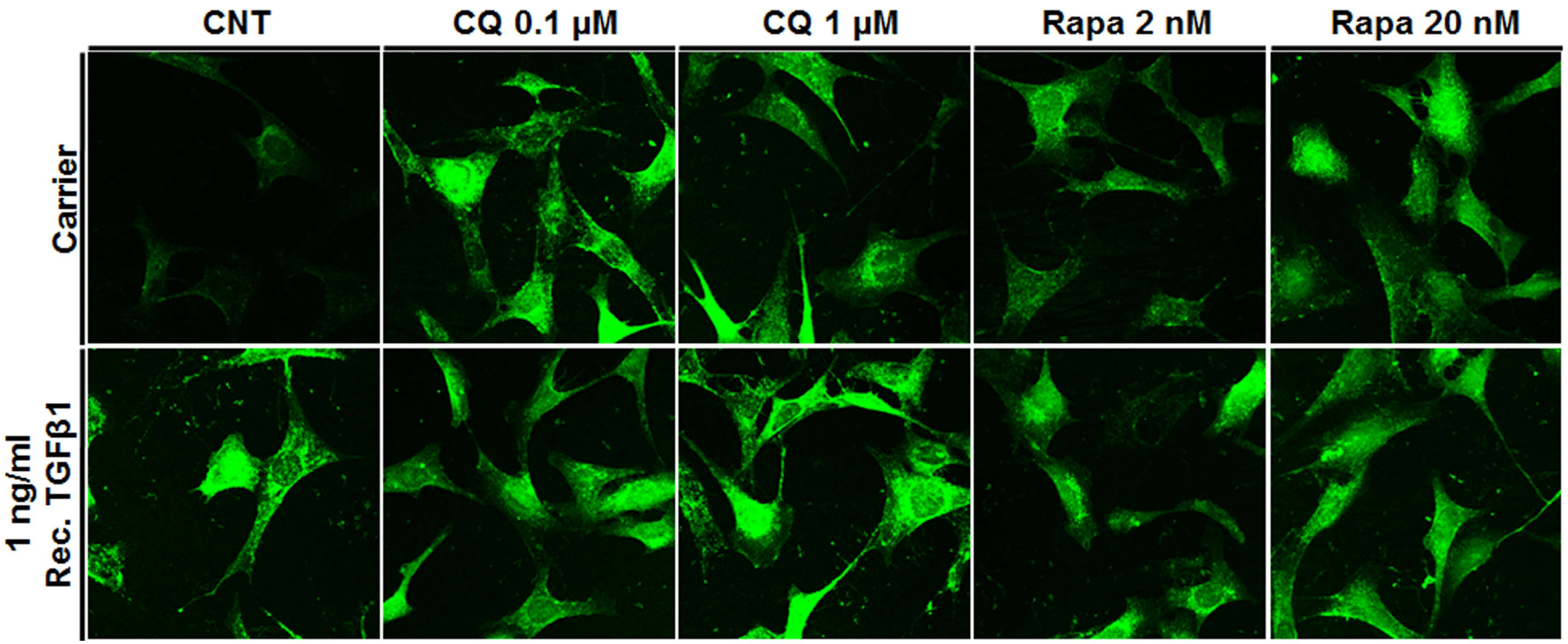

Figure S9

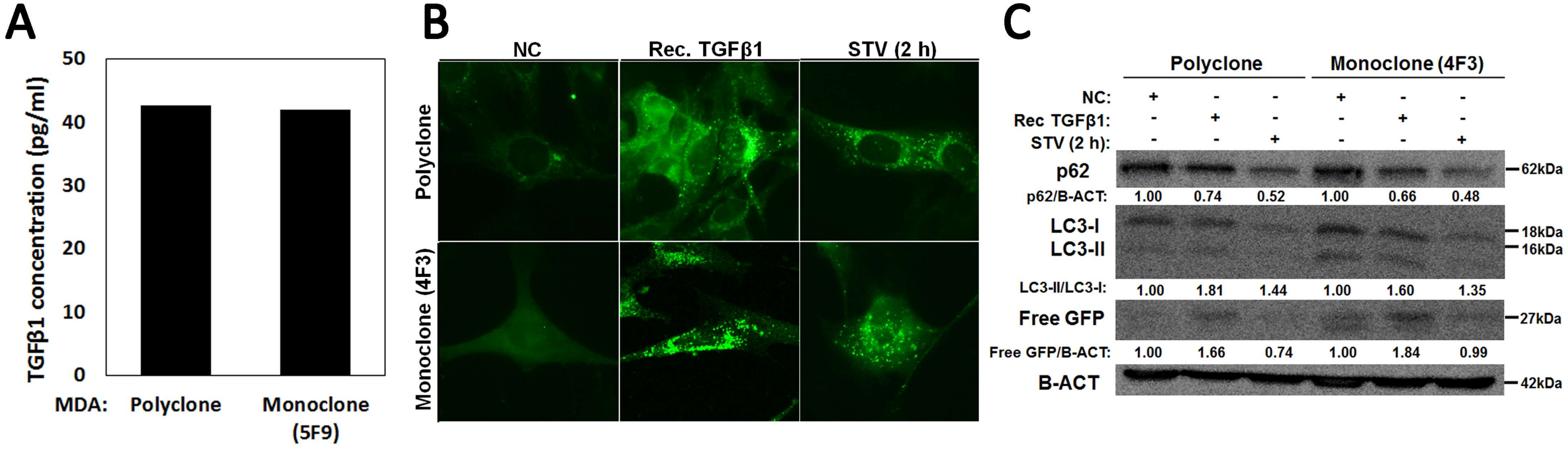

A
TGFβ1 concentration (pg/ml)
50
40
30
20
10
0
MDA:
Polyclone
Monoclone (5F9)
B
NC
Rec. TGFβ1
STV (2 h)
Polyclone
Monoclone (4F3)
C
Polyclone
Monoclone (4F3)
NC:
Rec TGFβ1:
STV (2 h):
p62
p62/B-ACT: 1.00 0.74 0.52 1.00 0.66 0.48
LC3-I
LC3-II
LC3-II/LC3-I: 1.00 1.81 1.44 1.00 1.60 1.35
Free GFP
Free GFP/B-ACT: 1.00 1.66 0.74 1.00 1.84 0.99
B-ACT
62kDa
18kDa
16kDa
27kDa
42kDa

Figure S10

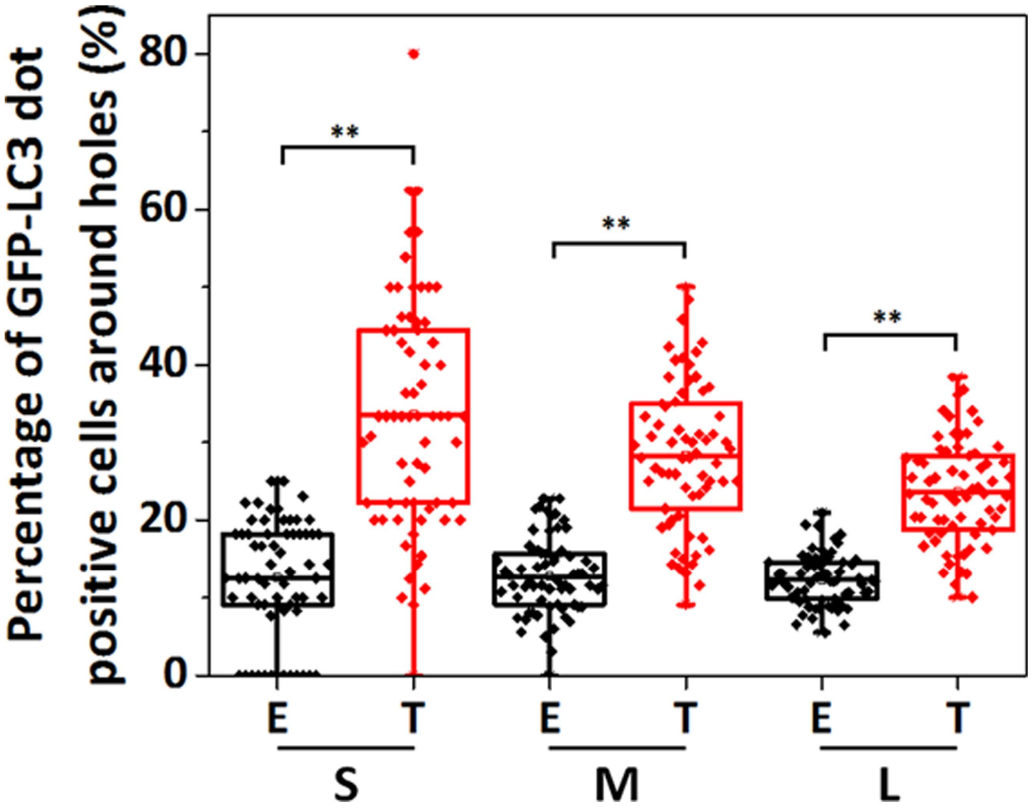
Percentage of GFP-LC3 dot positive cells around holes (%)
0
20
40
60
80
**
**
**
E
T
E
T
E
T
S
M
L

Figure S11

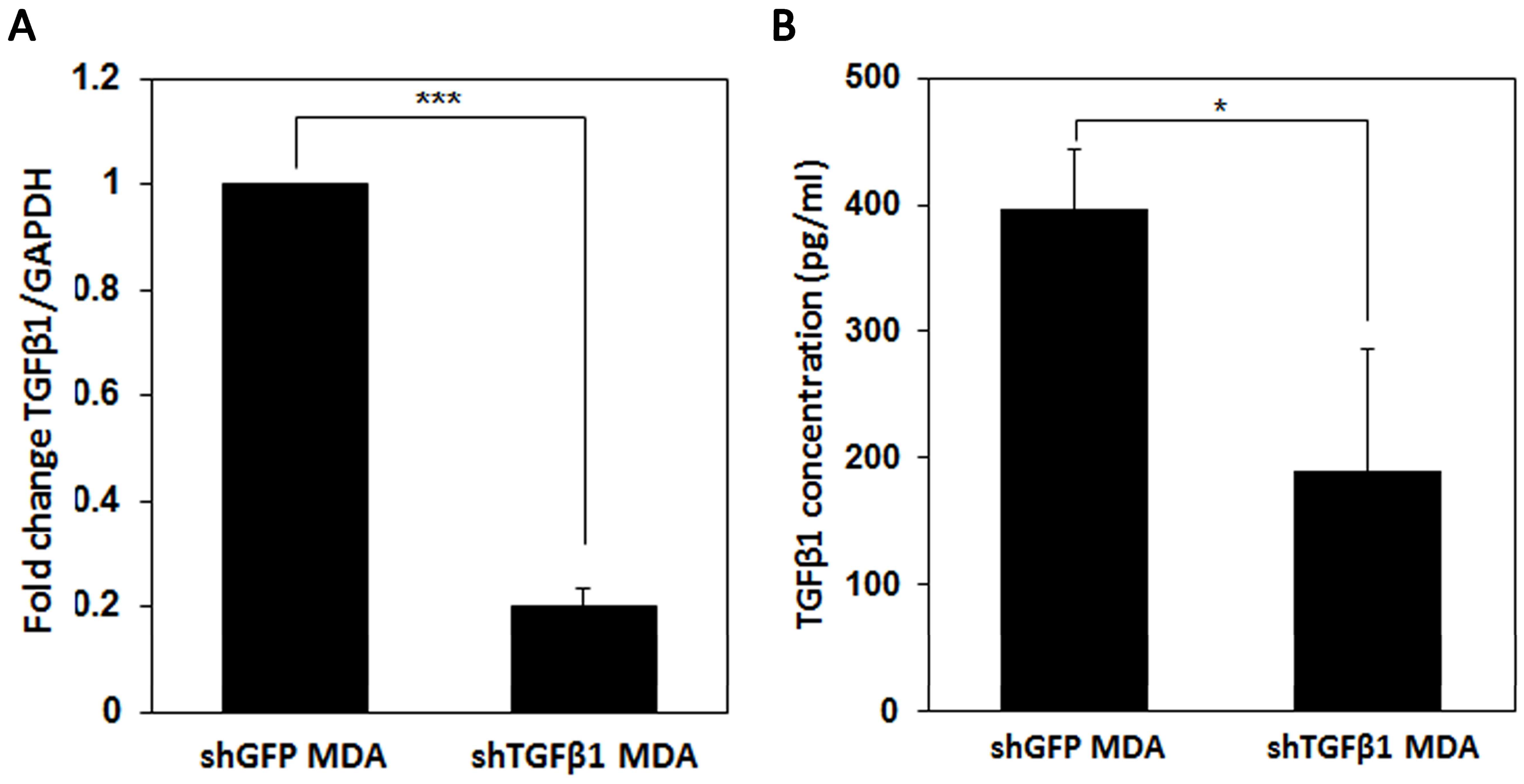
A
B
1.2
1
0.8
0.6
0.4
0.2
0
Fold change TGFβ1/GAPDH
***
shGFP MDA
shTGFβ1 MDA
500
400
300
200
100
0
TGFβ1 concentration (pg/ml)
*
shGFP MDA
shTGFβ1 MDA

Figure S12

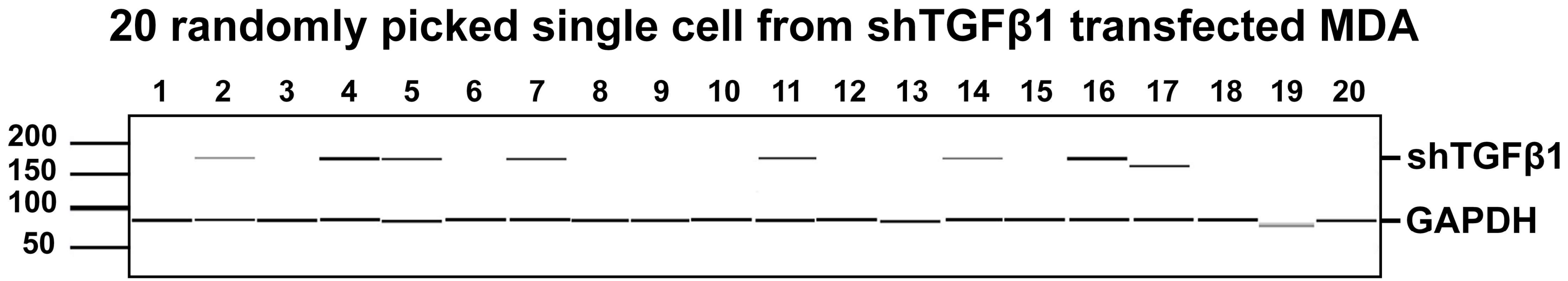
20 randomly picked single cell from shTGFβ1 transfected MDA
1 2 3 4 5 6 7 8 9 10 11 12 13 14 15 16 17 18 19 20
200
150
100
50
shTGFβ1
GAPDH
